# Virtual Reality–Assisted Therapy Compared to Cognitive Behavioral Therapy for Patients With Treatment-Resistant Schizophrenia: Assessor-Blind Randomized Controlled Trial

**DOI:** 10.2196/106791

**Published:** 2026-09-16

**Authors:** Mélissa Beaudoin, Stéphane Potvin, Sabrina Giguère, Charles-Édouard Giguère, Frederick Aardema, Amal Abdel-Baki, Luigi De Benedictis, Olivier Lipp, Pierre Lalonde, Emmanuel Stip, Alexandra Fortier, Kingsada Phraxayavong, Alexandre Dumais

**Affiliations:** 1 Department of Psychiatry and Addictology, Université de Montréal, 2900, boul. Édouard-Montpetit, Montreal, QC, H3T 1J4, Canada, 1 514-251-4000 ext 3925; 2 Institut universitaire en santé mentale de Montréal, Montreal, QC, Canada; 3 Centre hospitalier de l’Université de Montréal, Montreal, QC, Canada

**Keywords:** psychotherapies, digital interventions, treatment-resistant schizophrenia, virtual reality–assisted psychotherapy, randomized controlled trial

## Abstract

**Background:**

Auditory verbal hallucinations (AVH) are among the most disabling symptoms of schizophrenia and often persist despite treatment. Virtual reality–assisted therapies (VRTs) are a new generation of relational interventions for AVH, but comparative evidence against active interventions is currently lacking.

**Objective:**

This study aimed to assess whether VRT (9 sessions) is superior to a short course of cognitive behavioral therapy (CBT) targeting AVH (9 sessions) in reducing AVH in individuals with treatment-resistant schizophrenia.

**Methods:**

In this assessor-blind, parallel-group randomized controlled trial conducted from 2019 to 2026 at an academic center in Montreal (Canada), adults with schizophrenia or schizoaffective disorder and persistent AVH were either referred by their health care team or self-referred. A total of 136 participants were randomly assigned 1:1 to VRT or CBT, stratified by sex and clozapine use status. Both 9-session interventions targeted maladaptive beliefs and relationships with voices and were administered by trained psychotherapists. The predetermined primary outcome was the evolution of AVH severity over time, measured at baseline, post treatment, and 3 months post therapy using the auditory hallucination subscale of the Psychotic Symptoms Rating Scale. Secondary outcomes notably included the general psychotic symptomatology measured using the Positive and Negative Syndrome Scale. Linear mixed-effects models were used to assess time-by-treatment interactions. Psychotherapy sessions and assessments were conducted primarily in person, with CBT being occasionally delivered via videoconferencing during the COVID-19 pandemic.

**Results:**

Participants had a mean age of 40.3 (SD 12.8) years, 63.2% (86/136) were male, and 56.6% (77/136) received clozapine. Intention-to-treat analyses (VRT, n=67; CBT, n=69) showed a significant time-by-treatment interaction favoring VRT (*P*=.013) with a moderate effect size at 3 months post therapy (Cohen *d*=0.614). Both therapies showed significant within-group improvements in the primary outcome, with large effect sizes for VRT (Cohen *d*=0.81 post therapy and Cohen *d*=1.17 at 3 months) and moderate for CBT (Cohen *d*=0.58 post therapy and Cohen *d*=0.39 at 3 months). While there were no between-group differences for secondary outcomes, within-group improvements were observed in psychotic symptoms, emotional regulation, and voice acceptance for both therapies, and VRT also reduced maladaptive beliefs about voices and improved self-esteem.

**Conclusions:**

VRT outperformed a targeted short course of CBT in reducing persistent AVH for up to 3 months after the intervention in a North American population with treatment-resistant schizophrenia. Improvements were also seen in some secondary outcomes, such as general psychotic symptomatology, and those were similar for both interventions. Overall, these findings support the value of VRT as a personalized and clinically effective intervention.

## Introduction

Schizophrenia imposes a substantial and long-lasting burden across health, social, and economic domains [1]. A primary driver of this is treatment resistance, impacting around one-third of patients, for which clozapine is recommended [2,3]. However, tolerability is often a challenge, and some individuals still do not respond [4,5]. Considering these gaps, psychotherapy can be offered as an adjunct to help in coping with symptoms and enhance the quality of life [6,7]. Nevertheless, therapeutic options remain limited, underscoring the need to develop and validate adjunctive interventions.

Auditory verbal hallucinations (AVH) are highly prevalent and clinically distressing residual symptoms; therefore, psychotherapeutic frameworks often target them [8,9]. Notably, using a structured, goal-oriented approach aimed at reducing distress and anxiety, cognitive behavioral therapy (CBT) demonstrated low to moderate short-term effects on AVH severity and overall positive symptoms [2,10]. Nevertheless, its efficacy appears to be limited over time, highlighting the need to develop alternative nonpharmacological treatment modalities [11,12].

Novel psychological approaches have recently emerged, including relational therapies grounded in an interpersonal conceptualization of voice hearing, such as AVATAR (Audio Visual Assisted Therapy Aid for Refractory Auditory Hallucinations) therapy [13,14]. Through real-time digital animation and voice modulation, patients are invited to engage with a visual representation of a distressing voice, animated and controlled by a therapist. Initially developed in the United Kingdom, it has subsequently been adapted worldwide, including in Canada, through the integration of immersive 3D virtual reality (VR) [15-21]. The outcomes of the virtual reality–assisted therapy (VRT) have consistently demonstrated promising effects on AVH, affective symptoms, and beliefs about voices [9,16,17,20,21].

To date, most randomized controlled trials (RCTs) have evaluated AVATAR therapy against minimally active or inactive control conditions, such as supportive counseling or treatment as usual [15,18,19,21]. Consequently, whether it confers superiority over an established, evidence-based psychotherapy in reducing AVH severity remains an unresolved empirical question. The present trial addresses a critical gap in the literature as the first adequately powered assessor-blind RCT conducted exclusively in individuals with treatment-resistant schizophrenia and the first to compare VRT against a short course of CBT.

Hence, the primary objective of this trial was to determine whether VRT is superior to a short course of CBT adapted for AVH to serve as an active comparator (same duration and similar therapeutic targets) in reducing the impact of AVH in treatment-resistant schizophrenia for up to 3 months following the end of therapy. Secondary objectives included the evaluation of VRT’s potential superiority regarding other positive psychotic symptoms, general psychopathology, beliefs about and responses to voices, emotional regulation, self-esteem, and quality of life. Consistent with prior clinical evidence, the hypothesis was that VRT would demonstrate superiority over a short course of CBT in reducing AVH severity for up to 3 months after therapy.

## Methods

### Study Participants

Recruitment occurred between April 2019 and June 2025. Participants were either referred by their health care team or self-referred. Active efforts were made to inform health care providers about the project in 4 psychiatric institutions across the province, including the major psychiatric institutions on the island of Montreal, as well as in other urban and suburban areas, and online via videoconferencing. Presentations were also offered to patients and their support network through community organizations such as the *Société Québécoise de la Schizophrénie* and the *Association des parents et amis de la personne atteinte de maladie mentale*. Information about the project was available on a dedicated website, which included a self-referral form and contact information (phone number and email address) for more details on study eligibility. However, participants could come from anywhere in the province. The therapies were delivered at the *Institut universitaire en santé mentale de Montréal* located in Montreal, Canada. Participants were responsible for traveling to Montreal for therapy sessions. When needed, compensation for public transportation (eg, bus tickets) or taxi transportation was provided to facilitate attendance.

Participants were eligible if they were aged 18 years or older; diagnosed with either schizophrenia or schizoaffective disorder with persistent AVH; nonresponsive to 2 or more antipsychotic trials, each lasting more than 6 weeks at a therapeutic dosage equivalent to at least 600 mg of chlorpromazine daily; and on stable doses of medication during the 2 months before study enrollment with good adherence (defined as 80% or more of the doses being taken). Participants were excluded if they presented a neurological disorder, had an unstable and serious physical illness, had a substance use disorder in the past year, had received any form of CBT for psychosis in the past year, lacked the capacity to consent, or were currently experiencing an acute psychotic episode. These criteria were similar to those of other trials in the field of treatment-resistant schizophrenia [21-23]. Participants with insufficient French language proficiency to complete the study protocol were excluded. With the participant’s consent, attempts were made to contact each participant’s treating psychiatrist to confirm eligibility, inform them of their participation in the clinical trial, and ensure that no changes in psychiatric medication were planned in the near future. Moreover, participants could be excluded during therapy if their mental state deteriorated and prevented them from continuing the treatment.

All participants continued to receive standard psychiatric care throughout the study, which involved antipsychotic medication and regular outpatient psychiatric appointments. Eligible participants were randomly assigned in a 1:1 ratio to 1 of 2 parallel arms to receive either VRT or a short course of CBT.

### Ethical Considerations

The trial was conducted in accordance with the Declaration of Helsinki. It was approved by the institutional ethics committee of the *Centre intégré universitaire de santé et de services sociaux (CIUSSS) de l’Est-de-l’Île-de-Montréal* (project MP-12-2019-1726) in April 2019. Prior to their inclusion in the study, the information and consent form was carefully presented and explained to potential participants by a research assistant or psychiatric nurse. The paper form was also provided, along with time and space to make an informed decision. All questions were answered as needed, whether during the presentation or later by phone. Participants’ capacity to consent was also assessed; for example, participants were asked to summarize different sections of the form. If they agreed to participate and had the capacity to consent, they were allowed to provide written informed consent, and basic information was collected during the same visit, including sociodemographic data, contact information, and current use of clozapine (for randomization purposes). As compensation for their time, participants received CAD $20 (CAD $1=US $0.73 as of September 8, 2026) per clinical assessment.

### Design

#### Study Design and Oversight

The present study consisted of a randomized, controlled, assessor-blind, parallel-group clinical trial comparing 2 interventions: VRT and a short-course CBT protocol focused on AVH. The trial was conducted at an academic center (*Institut universitaire en santé mentale de Montréal*) located in Montreal, Canada. This clinical trial was registered on Clinicaltrials.gov (NCT04054778) on June 19, 2019. The CONSORT-eHEALTH checklist for RCTs is provided as Checklist 1.

Participants, after randomization, underwent 9 weekly, 1-hour sessions of either VRT or CBT for AVH. Clinical assessments were conducted approximately 1 week prior to the start of therapy (pretherapy), 1 week after the end of therapy (posttherapy), and 3 months after the last therapy session (3-month follow-up).

#### Randomization and Masking

After completion of the baseline assessment, participants were randomly assigned in a 1:1 ratio to VRT or CBT via an independent, secure, internet-based service sequence generation process [24], using randomly varying block sizes of 2, 4, and 6, stratified by sex and baseline clozapine treatment. An independent statistician generated the random allocation sequence, and no one else could access this sequence or the block size. Once a participant was deemed eligible, the study coordinator (KP) entered the participant’s sex and baseline clozapine treatment status into an internet-based randomization service, which generated the treatment allocation. The study coordinator then assigned the participant to the allocated intervention.

Because the treatment was psychotherapeutic, neither patients nor therapists could be blinded. Nevertheless, outcome assessments were conducted by masked research assistants (unaware of group allocation) who did not have access to the collected data. To prevent unmasking, assessments and therapy were conducted in different buildings, and participants were reminded not to disclose their treatment allocation at the beginning of each assessment. In the rare event of accidental unmasking, which the evaluators flagged as soon as it occurred, questionnaires and scales requiring clinical judgment were rated from audio recordings by an independent evaluator blinded to treatment allocation. To avoid interrater variation, this recording was performed across all assessment time points for the relevant participants.

### Therapies

#### VRT

VRT is a psychological intervention designed to treat individuals with treatment-resistant schizophrenia and persistent AVH by engaging them with a digital representation (“avatar”) of their most distressing hallucination. It was initially developed in the United Kingdom [19] and was adapted using VR by AD. Following the first pilot trial conducted by his research team, it was determined that 9 therapy sessions were optimal for maximizing outcomes and adequately consolidating learning [17]. Therefore, this therapy consists of 9 weekly 1-hour sessions.

The first session consisted of a case formulation focusing on the content and context surrounding the AVH, as well as the participants’ coping strategies. In doing so, the participants were invited to identify their most distressing voice and to imagine what it would look like. Then, participants were asked to create an avatar representing that voice. This step was conducted in a VR environment using an Oculus Rift head-mounted display. With the therapist’s support, participants customized their avatar’s physical appearance (eg, facial features, eyes, hair, skin color, and haircut) and voice characteristics (eg, pitch and volume) to closely match the voice they were hearing. This was done using a custom Unity 3D game engine, with unique avatars generated via the Morph3D Character System. The VR platform used was developed by OVA, a technology development company specializing in immersive VR and mixed reality. Finally, participants were asked to write down the content of their hallucinations on a typical day before the next session.

During subsequent sessions, participants were invited to engage in dialogue with their avatar in VR, which the therapist animated in real time. Playing the role of the avatar, the therapist spoke into a microphone, while their voice was transformed in real time using the Roland AIRA VT-3 voice transformer. Lip synchronization between the therapist’s speech and the avatar was achieved with the SALSA with RandomEyes Unity 3D extension. Facial expressions could also be modulated by the therapist; they were programmed to convey emotions using the Facial Action Coding System. The virtual environment consisted of a 3D avatar standing in a neutral single-colored cubic room, viewed from a first-person perspective. The participants and their therapist were separated by a one-way mirror, allowing the therapist to observe the participant during the session. Please refer to Figure S1 in Multimedia Appendix 1 for a visual of the setting in which the immersion took place.

Each therapeutic session (session 2 and onward) consisted of three components, each lasting approximately 5 to 20 minutes:

1. Preimmersion: this included a review of the previous week, review of the assignment (when applicable), and establishment of objectives for the current therapy session.
2. VR immersion: the participants engage in a dialogue with their avatars. In doing so, they are invited to experiment with different coping strategies.
3. Postimmersion: following immersion, the participant’s experience was debriefed, focusing specifically on the emotions that arose during the session. When applicable, assignments were then given for the upcoming week.

During the second and third sessions, participants were exposed to an avatar that faithfully reproduced their hallucinatory experience. For this purpose, the therapist facilitated a dialogue between the participant and the avatar using the sentences the participant had previously been asked to record, faithfully capturing the content. In return, participants were encouraged to respond to the avatar using their usual coping strategies, as well as new ones suggested by the therapist, to promote self-assertiveness. Patients were therefore provided with a safe, confidential therapeutic space to express their emotions, opinions, and demands. In doing so, participants were supported in challenging the perceived omnipotence of the voice. Starting in the third session, the avatar became more receptive and began to consider what the participant was telling them.

During the fourth session, the avatar became less confrontational or threatening and began to make attempts to reconcile with the participant, namely by encouraging the participant to verbalize their emotions and demands. It could notably support the participant and help them in suggesting new interpretations related to their hallucinatory experiences; for example, it would suggest that there might be a link between weak self-esteem and derogatory voice content. At the end of this session, participants were required to ask a close relative or friend about their qualities and list those for the next session.

For the fifth session, the focus formally shifted to self-esteem, which was reinforced by encouraging participants to reflect on their personal qualities. Using the list provided by the participants, they were encouraged to incorporate these positive attributes into the dialogue with their avatar, using self-worth to invalidate deprecating comments.

In the final consolidation sessions (sixth to ninth), participants were encouraged to apply what they had learned in the experiential sessions. The aim was to highlight the participants’ progress, promote relapse prevention, and support them in accepting and living with the residual voices. Participants were also guided in formulating aspirations for their future relationship with the voices as well as for moving forward with their lives.

Over the course of therapy, the therapist gradually modified the avatar’s speech and tone to align with the participant’s growing sense of empowerment, transforming the avatar from abusive to more helpful and supportive. Additionally, new suggestions regarding the participants’ beliefs about the voices and their origins were introduced. During the immersive sessions, the therapist could also pause at any point; make the avatar disappear; and intervene as themselves to guide, encourage, or assess the participant’s anxiety or fear levels.

Recordings of the immersive portion of each session were made available to participants during therapy, and they were strongly encouraged to listen to them between sessions.

#### Short-Course CBT for Auditory Verbal Hallucinations

The CBT therapy manual was developed collaboratively by one of the lead researchers, a CBT expert, and 2 psychologists with established proficiency and extensive clinical experience in delivering CBT. The manual was specifically adapted to serve as an active comparator to VRT, most notably by targeting the same primary outcome (AVH) and adhering to an equivalent short-course treatment format comprising 9 weekly, 1-hour sessions [10,25].

The first therapy session was devoted to case formulation, focusing on exploring the participant’s voice content, contextual factors, and strategies for managing AVH. The second session focused on psychoeducation and the normalization of hallucinatory experiences, including the use of well-known public figures who have reported similar phenomena, and the presentation of the vulnerability-stress model with concrete examples. AVH was also described and characterized, with particular attention to identifying triggers, contexts, and modulators associated with voice-hearing episodes. The third session focused on presenting the cognitive model of AVH, illustrated using a patient-specific example. Together with the patient, triggers were identified, followed by the anxiety-provoking thoughts and associated beliefs that ensued. The fourth session focused on learning about various attributional mechanisms. Through situational exercises and patient-generated examples, participants were encouraged to recognize that multiple interpretations can be drawn from the same event, thereby fostering greater cognitive flexibility. The fifth session focused on coping mechanisms. The therapist introduced multiple adaptation strategies (eg, behavioral distraction, cognitive distraction, and socialization) to help patients manage voice-hearing experiences. Patients were encouraged to reflect on which strategies they had attempted and to evaluate whether these strategies were effective in reducing distress. The sixth session focused on modifying beliefs and formulating alternative explanations. Using Socratic questioning, evidence evaluation, and behavioral experiments, the therapist supported the patient in generating more plausible alternative explanations for their AVH. Patients were encouraged to assess the likelihood of each interpretation through structured exercises, thereby testing and refining their beliefs. The seventh and eighth sessions focused on mindfulness-based exercises grounded in sensory awareness. Participants were instructed not to attempt to suppress the voices but rather to observe and listen to them without acting on them or judging them. Mindfulness practice was encouraged during actual voice-hearing episodes occurring during therapy or through guided recall of situations in which voices had been present. The final session focused on consolidating the skills and coping strategies acquired throughout therapy. A collaborative relapse prevention plan was developed with the participant, outlining individualized strategies for early identification and management of exacerbations.

Throughout therapy, various questionnaires and exercises were incorporated to facilitate learning. Moreover, participants were asked to complete homework between sessions, including required readings and a voice journal. This voice journal initially included when the voice occurred, the context, the intensity, and the associated emotions. As therapy progressed, participants were also invited to apply coping strategies and document their impact in this journal.

Although it was initially intended to be delivered in person like VRT, CBT was occasionally delivered via videoconferencing during the COVID-19 pandemic. Although data are still limited for schizophrenia, videoconferencing-delivered individual CBT is generally considered to be as effective as in-person one-on-one CBT for most psychiatric conditions, with recent meta-analyses showing noninferior outcomes between the 2 formats [26-28].

#### The Course of Therapy and Therapist Training

Both therapeutic modalities were manualized and had been delivered and investigated in previous randomized clinical trials [16,17]. Participants were provided access to a 24/7 helpline staffed by an unblinded psychiatric nurse, enabling them to discuss any concerns, adverse effects, or difficulties arising between sessions as needed. In addition, a psychiatric nurse called participants to remind them of the session the day before. Therapies were delivered by 3 psychologists, 1 psychiatrist with prior expertise in VRT, and 2 psychiatry residents under the supervision of the VRT expert. All therapists had prior clinical experience treating psychotic disorders (mean experience 12.75 [SD 8.2] years, range 1‐20 years), and therapist profiles—including professional background and years of experience—were balanced across the 2 treatment arms.

Training procedures encompassed direct observation of live therapy sessions, review of the therapy manual, and systematic listening to audio recordings of therapy sessions. Furthermore, each therapist’s initial cases were conducted under the direct supervision of either the VRT expert or the CBT expert, the investigators responsible for developing and delivering the respective interventions in the pilot randomized trial [16]. Psychiatry residents additionally received weekly individual psychotherapy supervision from the VRT expert throughout the duration of their therapy delivery.

Clinical Assessments

Standardized assessments were performed 1 week before the first therapy session (pretherapy), 1 week after the last session (posttherapy), and 3 months after the last therapy session (3-month follow-up) by a research assistant and nurses. While assessments were conducted primarily in person, they occasionally took place via videoconferencing during the COVID-19 pandemic.

First, sociodemographic data (eg, age, sex, ethnicity, education level, and income) were collected prior to the first formal assessment. This visit generally occurred approximately 1 or 2 weeks prior to the first clinical assessment.

Then, the first standardized assessment was conducted in 2 visits, generally 1 or 2 weeks prior to the first therapy session (7 days). During the first part of the pretherapy evaluation, the diagnosis of schizophrenia or schizoaffective disorder (as per DSM-5 [Diagnostic and Statistical Manual of Mental Disorders, Fifth Edition] criteria) was confirmed using the Structured Clinical Interview for the Diagnostic and Statistical Manual of Mental Disorders, Fifth Edition [29]. During the same visit, information was also collected on participants’ age at first contact with psychiatry and diagnoses.

The second part of the evaluation was blinded and included all quantitative questionnaires and scales assessing the primary and secondary outcomes of the study; it was identical to those conducted post therapy and at the 3-month follow-up. The predetermined primary outcome of the RCT was the overall severity of AVH, assessed using the total score of the auditory hallucination subscale of the Psychotic Symptoms Rating Scale (PSYRATS-AH) post treatment and at the 3-month follow-up. The PSYRATS-AH is a well-validated, dimensional, semistructured, assessor-rated clinical interview comprising 11 items that quantify multiple phenomenological dimensions of AVH, including frequency, loudness, voice-associated distress, and attribution (beliefs about the origin) [30]. This clinical tool has shown excellent interrater reliability (ICC=0.9) and good validity [30]. Of note, the PSYRATS-AH subscale was amended so that participants who no longer experienced hallucinations at the time of follow-up were assigned a total score of 0 for inclusion in the analyses.

Secondary outcomes were also assessed post treatment and at the 3-month follow-up. Delusions were assessed with the delusions subscale of the Psychotic Symptoms Rating Scale (PSYRATS-D), which includes measures about the frequency of delusions and delusion-associated distress [30]. Symptoms of schizophrenia were evaluated using the Positive and Negative Syndrome Scale (PANSS) [31]. This scale was separated into 5 symptom clusters using its demonstrated factorial structure: positive, negative, disorganization, depression-anxiety, and excitement [32]. Patients’ beliefs about their voices and related coping mechanisms were measured with the Beliefs About Voices Questionnaire-Revised (BAVQ-R), which includes 3 subscales assessing beliefs (ie, malevolence, benevolence, and omnipotence) and 2 subscales assessing the emotional and behavioral components of AVH (ie, resistance and engagement) [33]. Similar to the PSYRATS, participants who no longer experienced AVH at follow-up were assigned a BAVQ-R total score of 0. Acceptance-based attitudes and behaviors toward voice-hearing experiences were assessed with the Voices Acceptance and Action Scale (VAAS) [34]. Self-esteem was assessed using the Rosenberg Self-Esteem Scale (RSES), and the ability to understand, accept, and manage emotions effectively was assessed using the Difficulties in Emotion Regulation Scale (DERS-18) [35,36]. The quality of life and psychosocial functioning were assessed using the self-reported Quality of Life Enjoyment and Satisfaction Questionnaire Short Form (QLES-Q-SF) [37]. Additional details on all these tools, including their psychometric properties, are available in Table S1 in Multimedia Appendix 1.

All postrandomization evaluations were conducted by trained and experienced research professionals under blinded conditions. Assessors received training through interview videos and reading materials, with particular emphasis on instruments that require clinical judgment. To ensure interrater reliability, evaluators were trained together using standardized videotapes and consensus ratings. For each assessor, the first few assessments were conducted under the supervision of the lead researcher (AD), who provided constructive feedback. Regular team meetings were held to ensure good interrater reliability. To improve consistency, all quantitative assessments of a given participant were conducted by the same blinded evaluator.

The Negative Effects Questionnaire (NEQ), an instrument designed to assess adverse and unwanted effects of psychological treatments, was administered [38]. It includes 6 domains: symptoms, quality of the treatment, dependency on the treatment or therapist, stigma (ie, being perceived negatively by others because of the treatment), hopelessness, and lowered self-esteem [38,39]. Because of the particular risk of unblinding associated with this questionnaire, it was administered by an unblinded psychiatric nurse after the therapy. Potentially serious adverse events, including physical and psychiatric hospitalizations, were documented by an unblinded research nurse based on reports from the treatment team, participants, or family members.

Finally, assessment data from paper questionnaires were entered into a secure database by blinded research assistants. Data entry was performed twice independently to minimize transcription errors.

### Surveillance, Protocol Deviations, and Treatment Fidelity

Protocol deviations and undesirable treatment effects were compiled, analyzed, and presented by a graduate student (MB). Four categories of deviation were documented, including assessment timing, evaluator blinding, changes in antipsychotic medications, and treatment fidelity. The study was then overseen regularly by a steering committee that included the involved researchers, clinicians, a graduate student (MB), the president of the ethics committee, a biostatistician (CÉG), and one patient partner. After each meeting, the committee had to vote to continue the study, and the vote had to be unanimous to allow continuation. Moreover, the committee’s mandate was to recommend future adjustments to the study, notably to minimize and manage protocol deviations. If serious adverse events had occurred, a protocol was in place to ensure that they would have been handled seriously, including careful revision of the event by an independent committee to determine whether it was linked to therapy and what could have been done to prevent it. Moreover, the treatment fidelity was evaluated across 120 randomly selected sessions from both conditions, comprising 60 sessions from the CBT condition and 60 sessions from the VRT condition. Adherence to the respective treatment manuals was assessed using structured fidelity checklists developed specifically for each intervention. All items were rated dichotomously by an independent and trained evaluator. Fidelity ratings were conducted by 7 evaluators, comprising research assistants with clinical training, psychiatry residents, or graduate-level students. Evaluators received standardized training from the CBT and VRT experts, respectively, based on the corresponding treatment manuals. Finally, potential between-group differences in dropouts, exclusions, losses to follow-up, and overall completion rates were also examined.

### Statistical Analysis and Sample Size Calculations

Treatment efficacy and comparisons between treatments for primary and secondary outcomes were assessed using linear mixed models with restricted maximum likelihood (REML) estimation for missing data. Fixed effects included time (pretherapy, posttherapy, and 3-month follow-up), group (CBT and VRT), sex, clozapine use, and the interaction between time and group. A random intercept within subject was included, and residual variance was heterogeneous across time. Analyses were conducted according to the intention-to-treat principle, including all randomly assigned participants. Both between-group (treatment) and within-group (time) comparisons were examined, and time-by-treatment interactions were used to examine whether there was a significant change between VRT and CBT over time. Linear mixed models were chosen because they are well established and recommended for analyzing psychotherapy RCTs with repeated measures. They handle unequal variances and correlated data, which are very common in treatment trials, and allow unequal numbers of follow-ups, making them suitable for clinical trial data. Furthermore, linear mixed models are based on maximum likelihood methods rather than the usual ANOVA methods, which require data sphericity and are not robust with small to moderate sample sizes [40,41]. Post hoc false discovery rate corrections were applied separately within each questionnaire across all of its outcomes (eg, total score and subscale scores), excluding the prespecified primary outcome (PSYRATS-AH total score), to the between-group contrasts post therapy and at the 3-month follow-up, as well as to the within-group analyses [42]. These adjusted analyses were considered exploratory, as no multiplicity adjustment strategy was prespecified.

To evaluate whether there were significant differences in antipsychotic use between the 2 groups at baseline, posttherapy, and 3-month follow-up, doses were converted to olanzapine equivalents and analyzed using the Mann-Whitney *U* test. The number of participants with a medication change in each group was compared using the chi-square test. Moreover, intervals between assessments and therapy sessions were also compared between the 2 groups using the Mann-Whitney *U* test. Chi-square or Fisher tests (as appropriate) were performed to assess whether there were systematic differences between groups in dropout rates, exclusion, and missing data. Potentially serious adverse events were compared between treatment groups using Fisher exact test. Between-group differences in the NEQ total score and subscale scores were assessed using 2-tailed independent-samples *t* tests, and mean differences with 95% CIs were reported.

Drawing on findings from comparable studies, pilot data on the effects of VRT on AVH, and the small to moderate effect sizes typically observed for CBT, the between-group statistical power for the PSYRATS-AH outcome was estimated at *f*=0.25 [15,16,19,43]. Sample size calculations were performed using G*Power. With a 2-sided *α*=.05 and 80% power, a sample of 52 participants per arm was required to detect differences using pretreatment and posttreatment assessments. To account for an anticipated 20% attrition rate (based on our pilot trial) and 5 df for potential confounders, the final recruitment target was set at 136 participants [16].

Effect sizes (Cohen *d*) were categorized as small (0.2), medium (0.5), or large (>0.8) [44]. Effect sizes were estimated using R software (version 4.5.2; R Foundation for Statistical Computing) *emmeans* package (version 2.0.3), using the square root of the sum of the variances (random intercept + residuals) as sigma and the residuals df for the calculation [45]. Two-tailed *P* values were reported using .05 as the significance threshold. Analyses were performed independently by a doctoral student (MB) and an independent biostatistician with 15 years of experience (CÉG), using IBM SPSS Statistics for Windows (version 31) and R Statistics, (version 4.5.2; R Foundation for Statistical Computing), respectively, to ensure consistent results [46,47].

## Results

### Sample Characteristics

**Figure 1.**
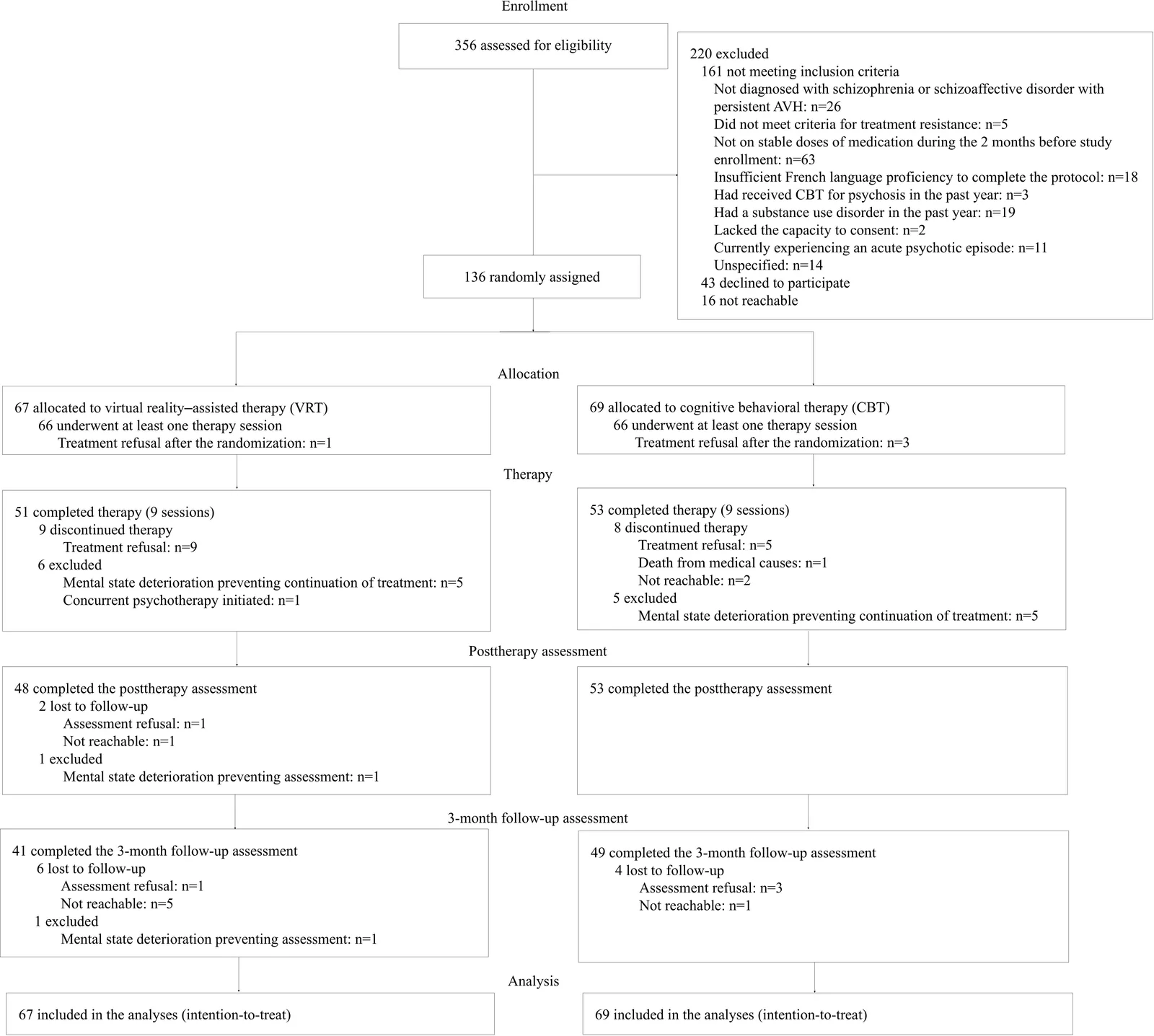
CONSORT diagram of all participants who were assessed for eligibility, randomized to virtual reality–based therapy or cognitive behavioral therapy, and completed therapy, posttherapy assessment, and 3-month follow-up assessment. AVH: auditory verbal hallucinations.

Participant recruitment occurred between April 2019 and June 2025. A total of 356 individuals were referred and assessed for eligibility across geographically and demographically diverse locations in Quebec, Canada. Of these, 136 participants met the inclusion criteria and were recruited and randomly assigned, yielding a recruitment rate of 38.2% (136/356; see Figure 1). Participants were recruited from 10 health regions across Quebec, representing both urban and mixed urban–rural catchment areas. Most participants were recruited from the 5 CIUSSSs on the island of Montreal (Centre-Sud, Centre-Ouest, Est, Ouest, and Nord; n=107), while additional participants were referred from Laval (n=3), Montérégie (n=6), Lanaudière (n=12), Laurentides (n=1), Estrie (n=2), and Quebec (n=1). Three participants had no documented referral region. Referring regions ranged from the Montreal area (approximately 15‐50 km) to more distant regions located up to approximately 260 km away.

The main reasons for exclusion were having unstable medication use during the 2 months before enrollment (n=63), not meeting the diagnostic criteria for schizophrenia or schizoaffective disorder with persistent AVH (n=26), having a substance use disorder within the past year (n=19), having insufficient French language proficiency to complete the study protocol (n=18), and experiencing an acute psychotic episode at the time of screening (n=11).

Following randomization, 67 participants were allocated to the VRT condition and 69 to the short-course CBT condition. Baseline characteristics were well balanced across both treatment arms. Randomization was stratified by sex and clozapine use to ensure comparability between groups.

Therapy sessions were delivered between April 2019 and October 2025, and the last 3-month follow-up assessment was completed in January 2026. A total of 51 participants completed 9 VRT sessions for a total of 459 therapy sessions. In comparison, 53 participants completed 9 CBT sessions, for a total of 477 therapy sessions.

No significant between-group differences were found in overall completion rate, dropouts, exclusions, and losses to follow-up at the posttherapy and 3-month follow-up assessments; please see Table S2 in Multimedia Appendix.

The initial demographic and medical profiles of study participants appeared to be well balanced between the 2 groups (see Table 1). Participants had a mean age of 40.3 (SD 12.8) years (range 18‐69 years) and were predominantly male (63.2%, 86/136), White (59.6%, 81/136), single (82.4%, 112/136), and without children (83.1%, 113/136). Social assistance was the primary source of income for 67.6% (92/136) of the participants, and 75.7% (103/136) reported annual incomes below CAD $20,000 (CAD $1=US $0.73 as of September 8, 2026), which is below the poverty threshold. Among participants, 65.4% (89/136) had a primary diagnosis of schizophrenia, and 34.6% (47/136) had schizoaffective disorder. The mean duration since first contact with psychiatry was 16.7 (SD 11) years, and 56.6% (77/136) of the participants were receiving clozapine.

**Table 1.** Baseline sociodemographic and clinical characteristics (N=136).

Characteristic	VRT^a^ (n=67)	CBT^b^ (n=69)
Age (years), mean (SD)	42.2 (13.1)	38.4 (12.2)
Sex assigned at birth, n (%)		
Male	42 (62.7)	44 (63.8)
Female	25 (37.3)	25 (36.2)
Ethnicity, n (%)		
White	40 (59.7)	41 (59.4)
Visible minorities	27 (40.3)	28 (40.6)
Marital status, n (%)		
Single	52 (77.6)	60 (87)
Divorced/separated	2 (3.0)	1 (1.4)
Widowed	1 (1.5)	0 (0)
Married/common law	8 (11.9)	5 (7.2)
In a relationship without cohabitation	3 (4.5)	2 (2.9)
Other	1 (1.5)	0 (0)
Unknown	0 (0)	1 (1.4)
Parental status, n (%)		
Yes	9 (13.4)	12 (17.4)
No	57 (85.1)	56 (81.2)
Unknown	1 (1.5)	1 (1.4)
Education, n (%)		
High school completed	48 (71.6)	44 (63.8)
Graduate degree completed	9 (13.4)	6 (8.7)
Unknown	0 (0)	1 (1.4)
Primary revenue source, n (%)		
Work or scholarships	8 (11.9)	14 (20.3)
Spouse or family	1 (1.5)	1 (1.4)
Retirement, pensions, annuities	7 (10.4)	0 (0)
Invalidity or unemployment insurance	5 (7.5)	2 (2.9)
Social assistance	44 (65.7)	48 (69.6)
Other	1 (1.5)	3 (4.3)
Unknown	1 (1.5)	1 (1.4)
Primary diagnosis (according to DSM-5)^c^, n (%)		
Schizophrenia	46 (68.7)	43 (62.3)
Schizoaffective disorder	21 (31.3)	26 (37.7)
Clozapine use, n (%)	38 (56.7)	39 (56.5)
Illness history		
Time since first contact with psychiatry (years), mean (SD)	17.5 (11)	16.1 (11)

a VRT: virtual reality–assisted therapy.

b CBT: cognitive behavioral therapy.

c DSM-5: Diagnostic and Statistical Manual of Mental Disorders, Fifth Edition.

### Between-Group Differences

For auditory hallucination severity, the intention-to-treat analysis using linear mixed models revealed a significant time-by-treatment interaction measured by PSYRATS-AH total score (*P*=.013), demonstrating VRT’s superiority over a short course of CBT in the primary outcome. Regarding specific time points, no differences were seen at the posttherapy evaluation (*P*=.809), whereas VRT was significantly more effective than a short course of CBT targeting AVH at the 3-month follow-up (Cohen *d*=0.614, 95% CI 0.085‐1.143, *P*=.022).

When examining the different subscales of the PSYRATS-AH scale, it was observed that only the frequency subscale showed greater improvements with VRT than with the short-course CBT (*P*=.031), with again no differences at the posttherapy evaluation (*P*=.142) but a significant difference at the 3-month follow-up visit (Cohen *d*=0.513, 95% CI 0.080‐0.947, *P*=.020). No significant between-group differences were observed in AVH-related distress, attribution, and loudness.

Regarding the delusions subscale of the PSYRATS, there was a significant time-by-treatment interaction in the distress subscale, demonstrating overall superiority of VRT over a short course of CBT over time (*P*=.011), although no differences were seen when examining specific time points.

Finally, no significant between-group differences were observed for all the other secondary outcomes, including overall psychotic symptomatology (measured using the PANSS, including the positive, negative, disorganized, excitation/hostility, and anxio-depressive subscales), beliefs about the voices (measured using the BAVQR, including the malevolence, benevolence, omnipotence, resistance, and engagement subscales), voices acceptance and action (measured using the VAAS), emotional regulation (measured using the DERS), self-esteem (measured using the RSES), and quality of life (measured using the QLESQ-SF).

Detailed between-group results for all linear mixed models, including time-by-treatment interactions, can be found in Table 2.

**Table 2.** Between-group differences in longitudinal effects of virtual reality–assisted therapy (VRT) vs cognitive behavioral therapy (CBT) on auditory hallucination severity and related clinical outcomes using linear mixed models (N=136).^a^

Outcome, scale, and subscale	Treatment interaction by time point								Time-by-treatment interaction
	Posttherapy				3-month follow-up				
	Cohen *d*	95% CI	*P* value	Adjusted *P* value	Cohen *d*	95% CI	*P* value	Adjusted *P* value	*P* value
Primary Outcome									
Auditory hallucinations									
PSYRATS-AH total 0‐44	0.062	−0.446 to 0.571	.809	N/A	0.614	0.085 to 1.143	.022	N/A	.013
Secondary outcomes									
Auditory hallucinations—subscales									
PSYRATS-AH^b^									
Distress 0‐20	0.019	−0.454 to 0.491	.937	.937	0.405	−0.069 to 0.879	.092	.125	.084
Frequency 0‐12	0.298	−0.102 to 0.699	.142	.466	0.513	0.080 to 0.947	.020	.080	.089
Attribution 0‐8	0.066	−0.322 to 0.454	.736	.937	0.384	−0.068 to 0.836	.094	.125	.031
Loudness 0‐4	0.257	−0.168 to 0.682	.233	.466	0.214	−0.183 to 0.612	.288	.288	.195
Delusions									
PSYRATS-D^c^									
Total 0‐24	0.164	−0.224 to 0.551	.404	.601	0.032	−0.414 to 0.351	.870	.870	.085
Distress 0‐8	0.221	−0.151 to 0.593	.241	.601	0.262	−0.678 to 0.155	.215	.577	.011
Frequency 0‐16	0.050	−0.340 to 0.440	.800	.800	0.166	−0.210 to 0.541	.385	.577	.376
Psychotic symptomatology									
PANSS^d^									
Total 30‐120	0.120	−0.252 to 0.492	.525	.847	0.179	−0.255 to 0.614	.415	.522	.855
Positive 4‐28	0.144	−0.207 to 0.495	.417	.847	0.170	−0.184 to 0.524	.343	.522	.787
Negative 6‐42	0.333	−0.054 to 0.720	.090	.538	0.409	0.004 to 0.814	.047	.280	.924
Disorganized 3‐21	0.0917	−0.252 to 0.435	.598	.847	0.161	−0.247 to 0.569	.435	.522	.745
Excitation/hostility 4‐28	0.036	−0.337 to 0.409	.848	.847	0.289	−0.129 to 0.706	.173	.518	.504
Anxio-depressive 3‐21	0.070	−0.298 to 0.439	.706	.847	0.107	−0.270 to 0.485	.575	.575	.634
Beliefs about voices									
BAVQR^e^									
Total 0‐105	0.398	0.020 to 0.776	.038	.076	0.339	−0.001 to 0.680	.050	.099	.157
Malevolence 0‐18	0.051	−0.355 to 0.458	.803	.895	0.116	−0.259 to 0.491	.542	.782	.676
Benevolence 0‐18	0.465	−0.107 to 0.822	.011	.032	0.440	−0.089 to 0.792	.014	.041	.281
Omnipotence 0‐18	0.145	−0.217 to 0.507	.429	.644	0.086	−0.263 to 0.434	.627	.752	.073
Resistance 0‐27	0.025	−0.354 to 0.405	.895	.895	0.048	−0.320 to 0.417	.797	.797	.956
Engagement 0‐24	0.464	−0.128 to 0.800	.007	.032	0.471	−0.131 to 0.811	.007	.039	.294
Voices Acceptance and Action									
VAAS^f^ total 31‐124	0.177	−0.171 to 0.525	.316	N/A	0.010	−0.379 to 0.399	.960	N/A	.356
Emotional regulation									
DERS^g^ total 18‐90	0.086	−0.273 to 0.446	.636	N/A	0.025	−0.336 to 0.385	.893	N/A	.451
Self-esteem									
RSES^h^ total 0‐40	0.110	−0.237 to 0.458	.531	N/A	0.016	−0.354 to 0.385	.933	N/A	.667
Quality of life enjoyment and satisfaction									
QLESQ-SF^i^ total 14‐70	0.151	−0.247 to 0.549	.454	N/A	0.123	−0.211 to 0.456	.468	N/A	.383

a Data are raw mean scores with SD. Linear mixed models with maximum likelihood estimation were used. Cohen *d* are presented in absolute values. 95% CIs for Cohen *d* (min-max values) are given. Adjusted *P* values were calculated using the false discovery rate (FDR) procedure. For each questionnaire, the maximum possible score range is indicated.

b PSYRATS-AH: auditory hallucination subscale of the Psychotic Symptoms Rating Scale.

c PSYRATS-D: delusions subscale of the Psychotic Symptoms Rating Scale.

d PANSS: Positive and Negative Syndrome Scale.

e BAVQ-R: Beliefs About Voices Questionnaire-Revised.

f VAAS: Voices Acceptance and Action Scale.

g DERS-18: Difficulties in Emotion Regulation Scale.

h RSES: Rosenberg Self-Esteem Scale.

i QLES-Q-SF: Quality of Life Enjoyment and Satisfaction Questionnaire Short Form.

### Within-Group Effects

**Figure 2.**
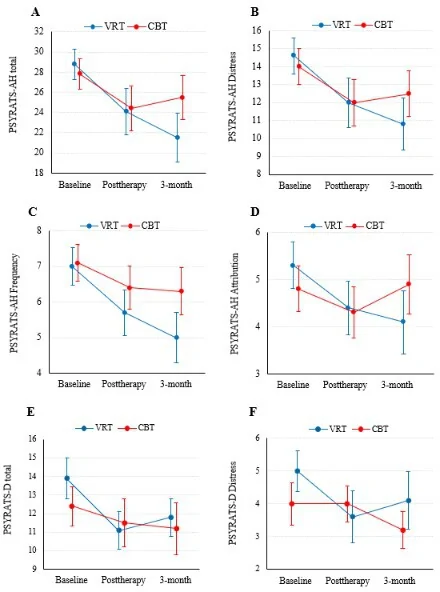
Primary and key secondary outcomes. The mean scores and 95% CIs, estimated using linear mixed models, are presented for 3 time points (baseline, posttherapy, and 3-month follow-up). The total hallucination score (A) is presented as well as the scores for each subscale, including distress (B), frequency (C), and attribution (D). For delusions, the total score (E) and distress subscale score (F) are presented. CBT: cognitive behavioral therapy; PSYRATS-AH: auditory hallucination subscale of the Psychotic Symptoms Rating Scale; PSYRATS-D: delusions subscale of the Psychotic Symptoms Rating Scale; VRT: virtual reality–assisted therapy.

Within-group effects of both therapies were also examined using linear mixed models. These results are visually presented in Figure 2, and detailed statistics can be found in Table S3 in Multimedia Appendix 1.

VRT produced a large effect on auditory hallucination severity immediately post therapy (Cohen *d*=0.796, *P*<.001) and at the 3-month follow-up (Cohen *d*=1.160, *P*<.001). Regarding specific auditory hallucination subscales, VRT demonstrated substantial and enduring improvements in hallucination-related distress, frequency, attribution, and loudness. For the delusions subscale, improvements were seen in the total score both post therapy and at the 3-month follow-up. Improvements were also seen in general psychotic psychopathology, as measured with the PANSS, at both time points, including the positive (both time points), negative (3-month follow-up only), and disorganized (posttherapy evaluation only) subscales. As for beliefs about the voices, significant improvements were also seen in the total score (both time points) as well as in the malevolence (3-month follow-up only) and omnipotence (both time points) subscales. Statistically significant improvements were also seen in voices acceptance and action (3-month follow-up only), emotional regulation (both time points), and self-esteem (both time points). No significant improvements were seen in quality of life enjoyment and satisfaction measured using the QLESQ-SF.

Finally, the short course of CBT targeting AVH showed significant improvements regarding auditory hallucination severity at the posttherapy visit only (Cohen *d*=0.572, *P*=.004), whereas no significant improvements were seen at the 3-month follow-up (*P*=.080). Nonetheless, significant improvements were seen in some subscales, including AVH-related distress (posttherapy only) and frequency (both time points). No significant effects were seen on attribution and loudness. Moreover, no improvements were seen in the delusions subscale of the PSYRATS. CBT had significant within-group impacts on overall psychotic psychopathology at all time points, including improvements in positive, negative, and disorganized symptoms. Regarding beliefs about the voices, although no significant changes were seen in the total BAVQR score, significant improvements were observed in the malevolence subscale only (both time points). Improvements were also seen in voices acceptance and action (all time points), emotional regulation (3-month follow-up only), and self-esteem (posttherapy evaluation only). No changes were seen in quality of life.

### Undesirable Effects

The NEQ was completed by 100 of the 136 participants (73.5% completion rate among those who underwent at least one therapy session). Missing data mainly occurred when participants were unreachable, which was also common among excluded participants and those lost to follow-up. Although efforts were made to contact excluded participants once their mental state stabilized, the questionnaire was not administered if they were unable to properly recall therapy. Reported negative effects were low and comparable across study conditions. The between-group comparison of the NEQ total score and subscale scores showed a significant difference only for the perceived treatment quality subscale, with participants reporting lower satisfaction with CBT than with VRT. Results by subscale are presented in Table 3. Additional information is presented in Figure S2 in Multimedia Appendix 1.

Other potentially serious adverse events were thoroughly examined. Hospital admissions were infrequent but more prevalent in the CBT group (6 admissions for psychological health events and 1 admission for a physical health event in the CBT group [the latter occurred in a participant who also had a psychiatric admission] vs only 2 psychiatric admissions in the VRT group). No temporal pattern was observed, as hospital admissions occurred after different therapy sessions, and hospitalization rates did not differ significantly between the 2 groups (*P*=.275). One detox admission occurred in the CBT group. Finally, one death occurred in the CBT group due to medical causes unrelated to the trial protocol.

**Table 3.** Mean frequency of reported therapy-related negative effects and number of affected participants by treatment group and Negative Effect Questionnaire subscale (n^a^=100).

Negative effects attributed partially or entirely to the treatment (by subscale)	VRT^b^ (n=50)		CBT^c^ (n=50)		Between-group comparison	
	Frequency, mean (SD)	Participants reporting effects, n (%)	Frequency, mean (SD)	Participants reporting effects, n (%)	Mean difference	95% CI^d^
Symptoms (10 items)	1.6 (2)	25 (50)	1 (2)	16 (32)	−0.48	−4.05 to 3.08
Quality of treatment (11 items)	0.9 (1.3)	20 (40)	1.9 (2.5)	25 (50)	3.51	0.02 to 7.01
Dependency (3 items)	0.3 (0.7)	11 (22)	0.2 (0.6)	7 (14)	0.17	−1.27 to 1.61
Stigma (2 items)	0 (0.2)	2 (4)	0.1 (0.3)	7 (14)	0	−3.40 to 3.40
Hopelessness (3 items)	0.2 (0.6)	8 (16)	0.4 (0.9)	10 (20)	1.63	−2.61 to 5.86
Feelings of failure (3 items)	0 (0.2)	2 (4)	0.2 (0.4)	2 (4)	1.56	−4.17 to 7.28
Total (32 items)	3.1 (3.4)	31 (62)	3.8 (5.1)	31 (62)	4.35	−3.08 to 11.79

a n: number of participants reporting effects.

b VRT: virtual reality–assisted therapy.

c CBT: cognitive behavioral therapy.

d 95% CI for mean intensity.

### Protocol Deviations

Protocol deviations were prospectively recorded and reviewed during steering committee meetings.

First, deviations in assessment timing were generally low. The interval between the initial session and the baseline assessment exceeded the 7-day threshold for a greater proportion of participants in the VRT group; similarly, a greater number of individuals in the CBT group required more time to complete the 3-month follow-up assessment. The other intervals were comparable, without statistically significant differences. Full details are provided in Table S4 in Multimedia Appendix 1.

Second, evaluator blinding was compromised in 3 posttreatment assessments, in which case outcomes requiring clinical judgment were rerated from audio recordings by an independent blinded evaluator for those participants.

Third, changes in antipsychotic medication were common, occurring in 24.8% of participants during therapy and in 50% of participants by the 3-month follow-up visit. However, no between-group differences were observed (please refer to Table S5 in Multimedia Appendix 1 for details). In addition, the olanzapine-equivalent dosage in antipsychotic medication did not differ between the 2 treatment arms at any of the 3 assessments, as detailed in Table S6 in Multimedia Appendix 1. Although the analyses followed an intention-to-treat approach, follow-up data, including outcome measures, could not be obtained for participants who were excluded, despite repeated attempts to recontact them at the time of exclusion. In most cases, exclusion occurred because participants were deemed too clinically unstable to continue therapy, which also generally prevented them from providing informed consent for a research evaluation. All available information, including baseline assessments, was nevertheless incorporated into the linear mixed model analyses.

### Treatment Fidelity

Results indicated good adherence to the study protocol across both conditions. In the VRT group, therapists achieved a mean fidelity rate of 96.8% (SD 3.8%; range 90%‐100%), while therapists in the CBT condition demonstrated a mean fidelity rate of 93.2% (SD 6.5%; range 81%‐100%).

## Discussion

This assessor-blind RCT aimed to determine whether VRT was superior to a short-course CBT in reducing AVH for up to 3 months after therapy. To our knowledge, this is the first fully powered RCT to compare an active psychotherapy (CBT) to VRT in patients with treatment-resistant schizophrenia. Relative to a short course of CBT targeting AVH (same length as VRT), VRT produced significantly greater reductions in overall AVH severity (*P*=.013), with superior benefits observed up to the 3-month follow-up visit (Cohen *d*=0.614, *P*=.022), indicating maintained benefits specific to VRT in the short to medium term. These findings support the superiority of VRT over CBT in reducing AVH severity, thereby confirming the primary hypothesis. This pattern aligns with findings typically reported in CBT trials, in which reductions in hallucinations tend to be modest and often wane at follow-up [10].

At posttreatment, no significant between-group difference was observed between VRT and CBT, contrasting with previous RCTs that reported greater reductions in AVH severity with VRT compared with supportive counseling, treatment as usual, or enhanced treatment as usual with supportive counseling [15,18,21]. This discrepancy may partly reflect differences in control conditions, as CBT is an active intervention with demonstrated efficacy for AVH [9,10]. Consistent with this interpretation, a recent meta-analysis found that the effects of VRT varied according to the type of control intervention [48]. Conversely, at midterm follow-up, VRT showed greater improvement than CBT, whereas previous RCTs did not find significant differences between VRT and their respective control conditions [15,18,21]. In the present trial, this difference may reflect sustained improvements in the VRT group alongside a lack of continued improvement in the CBT group, whereas control groups in previous trials continued to improve during follow-up. This finding is consistent with a recent meta-analysis reporting comparable effects of VRT and CBT on AVH severity at posttreatment but greater efficacy of VRT at 3-month follow-up [49]. In addition, half of the present sample was taking clozapine, consistent with an ultraresistant population [50], a subgroup in which CBT shows limited durability in symptom improvement [11]. The proportion of ultraresistant participants was higher than in the AVATAR and Challenge trials, reflecting differences in treatment resistance profiles between our RCT and previous RCTs.

Regarding secondary outcomes, significant between-group effects were seen in the attribution of auditory hallucinations, which could indicate that participants changed their perspective regarding the origin of the voice, consistent with studies describing the experience of participants for whom the attribution of the voice shifted completely from an external to an internal source, resulting in a change of avatar that ended up representing themselves [51,52]. Moreover, although it was not the main target of therapy, VRT showed greater reductions in delusion-associated distress compared to CBT, possibly because these delusions were often tied to the voices, whose features (content, frequency, and associated beliefs) are amenable to therapeutic modification. Finally, although within-group improvements were seen, no significant between-group effects have been observed for the other secondary outcomes, including general psychotic psychopathology, beliefs about the voices, voices acceptance and action, emotional regulation, self-esteem, and quality of life. This lack of differences does not appear to be explained by a lack of efficacy but rather a similar effectiveness for both interventions in many of these outcomes, including the general psychotic psychopathology measured using the PANSS positive symptoms subscale, which showed significant within-group improvements for both therapies. Indeed, although CBT has previously been shown to reduce AVH with only a low to moderate efficacy [9,10], it remains highly recommended for the treatment of residual positive symptoms [53,54]. Regarding within-group effects, VRT showed significant improvements on all auditory hallucination subscales, including voice-associated distress, frequency, and attribution. These findings suggest that VRT not only helped participants manage their emotions but also had an impact on how frequent and disturbing the hallucinations could be. Significant effects were observed on voice-related beliefs, specifically omnipotence, as well as on voice acceptance, indicating broader shifts in the relational schema toward voices. Furthermore, VRT was associated with significant reductions in psychotic symptoms, as well as improvements in self-esteem and emotional regulation. The changes observed in psychotic symptoms (PANSS total and positive subscale scores) are consistent with the existing psychotherapeutic literature on psychosis (eg, [55]). These findings highlight the meaningful psychological benefits of VRT, consistent with most previous trials [16,18].

This study has several notable strengths. Foremost, it represents one of the largest RCTs to date evaluating VRT and comparing it with a targeted short course of an active, evidence-based comparator (CBT for AVH). Indeed, CBT represents the psychosocial intervention with the strongest demonstrated efficacy for the treatment of positive symptoms in schizophrenia and is endorsed by major clinical practice guidelines, rendering it an appropriate active comparator and providing a robust test of relative efficacy [2]. It should nevertheless be noted that the limited number of CBT sessions, chosen to be comparable to VRT, could have impacted its effectiveness [56]. Moreover, the trial specifically targeted treatment-resistant populations, including almost 60% of participants receiving clozapine, enhancing the clinical relevance of the findings for a group that is harder to treat and for whom therapeutic options are more limited. The proportion of participants presenting AVH despite receiving clozapine at an adequate dose (ie, ultraresistant participants) was markedly higher than the approximately 25% reported in comparable trials in this area [18,21]. This is an important consideration, given that the existing literature suggests that the benefits of psychosocial interventions are generally less pronounced when delivered to this population [12]. The observed differential between VRT and CBT is therefore particularly significant, as it was demonstrated in a sample representing one of the most clinically challenging populations within the schizophrenia spectrum.

Despite numerous strengths, a few limitations must be acknowledged. First, due to the COVID-19 pandemic, CBT was delivered remotely to 35.8% of patients. In contrast, VRT remained in person, as this modality required digital materials and could not be delivered remotely. This discrepancy in delivery format may have affected the comparability of the interventions. However, the primary outcome scores (PSYRATS-AH) did not differ significantly between those who received in-person vs remote CBT at posttherapy (*P*=.709) and at the 3-month follow-up (*P*=.919). In addition, meta-analyses have demonstrated that CBT is equally effective when delivered in person or remotely, suggesting that this modality change is unlikely to have substantially affected the study outcomes [26]. Second, while the COVID-19 pandemic also did not increase the number of participants with protocol deviations, the longest delays between assessments were observed during this period for both study arms. To minimize these delays, some evaluations were conducted remotely via videoconferencing. The pandemic also slowed down recruitment, particularly in 2020, leading to delays in the completion of the study. Third, although patients and their treatment teams were consulted to identify planned medication changes up to the posttherapy assessment for patient eligibility, unplanned medication changes still occurred in a subset of patients (22.9% for VRT and 26.4% for CBT). However, the proportion of patients who experienced medication changes did not differ significantly between the 2 groups. Additionally, these changes reflect typical real-world clinical practice and may enhance the generalizability of the findings. Fourth, another limitation concerns the controlled nature of the trial, as eligibility criteria and the number of therapy sessions may be adapted to individual clinical needs in routine practice. Indeed, these strict inclusion criteria could impact the generalizability of these findings, which should be considered in light of the study population, which was restricted to individuals with schizophrenia or schizoaffective disorder experiencing treatment-resistant AVH. This population was selected because these participants were determined to be the most likely to benefit from this intervention, as symptoms remain despite an already optimized pharmacological treatment. Because this population is more difficult to treat, the presented results are conservative. Nonetheless, recent studies conducted by other research groups have reported positive outcomes after extending avatar therapy to broader populations with psychotic disorders, suggesting that the intervention may be applicable beyond the specific population included in the present study [18,21]. Fifth, it should be noted that there could be selection bias in recruitment as a substantial proportion of referees declined (20%) or were unreachable (7%). Nonetheless, this could be representative of a clinical population undergoing psychotherapy since similar refusals and failure to reach patients can also occur in clinical practice. Finally, similar to most previous trials evaluating the effectiveness of VRT, the study population was mostly Western and White. In addition, despite efforts to recruit from different inpatient and outpatient services from urban and suburban areas, therapy sessions were conducted in Montreal, thereby decreasing diversity. Nevertheless, although the sample size was limited, a study conducted in China showed good within-group efficacy without between-group superiority of VRT over CBT [20]. These results suggest that VRT could also be effective in an Asian population, although its potential superiority over CBT would require further adequately powered studies.

In conclusion, in the current trial, VRT demonstrated statistically significant superiority over an equivalent short course of CBT on overall auditory hallucination severity, auditory hallucination attribution, and delusion-related distress, while showing benefits equivalent to CBT across most symptom domains, including general psychopathology, voice beliefs, and emotional regulation. Unlike previous large-scale RCTs in this area, the present study compared VRT directly with a significantly active reference treatment (CBT), thereby providing a more stringent test of its relative efficacy. Future studies should incorporate extended follow-up periods to assess whether the benefits of VRT are sustained over time. Moreover, similar interventions or techniques using VR could also be developed or integrated into this version of VRT in order to tackle the other components of psychosis, such as delusions, social skills, or negative symptoms. Further research into the mechanisms of action underlying VRT is needed to identify its active therapeutic components and inform ongoing intervention refinement. Further analyses will also allow for the identification of subgroups of patients who respond better to one therapy or the other, which could be useful for selecting which therapy is most likely to benefit each patient from a personalized medicine perspective. Health economic analyses will also be essential for evaluating the cost-effectiveness of VRT and supporting its potential adoption within publicly funded mental health services. Finally, implementation projects are warranted to assess the feasibility and scalability of VRT across diverse real-world clinical settings, thereby facilitating its adoption in routine care pathways.

## Supplementary material

10.2196/106791 Multimedia Appendix 1 Figures depicting the setting of immersive sessions and number of patients per treatment group reporting each item on the Negative Effects Questionnaire, as well as tables showing between-group comparisons, within-group longitudinal changes, protocol deviations, equivalent olanzapine dosage at 3 time points, and evolution of feeling of presence over the course of virtual reality–assisted therapy.

10.2196/106791 Checklist 1 The CONSORT-eHEALTH checklist (V 1.6.1).

## Abbreviations

AVATAR: Audio Visual Assisted Therapy Aid for Refractory Auditory Hallucinations
AVH: auditory verbal hallucinations
BAVQ-R: Beliefs About Voices Questionnaire-Revised
CBT: cognitive behavioral therapy
CIUSSS: Centre intégré universitaire de santé et de services sociaux
DERS: Difficulties in Emotion Regulation Scale
DSM-5: Diagnostic and Statistical Manual of Mental Disorders, Fifth Edition
NEQ: Negative Effects Questionnaire
PANSS: Positive and Negative Syndrome Scale
PSYRATS: Psychotic Symptoms Rating Scale
PSYRATS-AH: auditory hallucination subscale of the Psychotic Symptoms Rating Scale
PSYRATS-D: delusions subscale of the Psychotic Symptoms Rating Scale
QLESQ-SF: Quality-of-Life Enjoyment and Satisfaction Questionnaire Short Form
RCT: randomized controlled trial
REML: restricted maximum likelihood
RSES: Rosenberg Self-Esteem Scale
VAAS: Voices Acceptance and Action Scale
VR: virtual reality
VRT: virtual reality–assisted therapy
