# Supplementary material for: Virtual Reality–Assisted Therapy Compared to Cognitive Behavioral Therapy for Patients With Treatment-Resistant Schizophrenia: Assessor-Blind Randomized Controlled Trial
Source: JMIR Ment Health. 2026 Sep 16;13:e106791. doi: 10.2196/106791 (PMC13580698; doi:10.2196/106791)
Supplement: Supplementary file 1 [file mental-v13-e106791-s002.pdf]

CONSORT-EHEALTH (V 1.6.1) - Submission/Publication Form

The CONSORT-EHEALTH checklist is intended for authors of randomized trials evaluating web-based and Internet-based applications/interventions, including mobile interventions, electronic games (incl multiplayer games), social media, certain telehealth applications, and other interactive and/or networked electronic applications. Some of the items (e.g. all subitems under item 5 - description of the intervention) may also be applicable for other study designs.

The goal of the CONSORT EHEALTH checklist and guideline is to be
a) a guide for reporting for authors of RCTs,
b) to form a basis for appraisal of an ehealth trial (in terms of validity)

CONSORT-EHEALTH items/subitems are MANDATORY reporting items for studies published in the Journal of Medical Internet Research and other journals / scientific societies endorsing the checklist.

Items numbered 1., 2., 3., 4a., 4b etc are original CONSORT or CONSORT-NPT (non-pharmacologic treatment) items.
Items with Roman numerals (i., ii, iii, iv etc.) are CONSORT-EHEALTH extensions/clarifications.

As the CONSORT-EHEALTH checklist is still considered in a formative stage, we would ask that you also RATE ON A SCALE OF 1-5 how important/useful you feel each item is FOR THE PURPOSE OF THE CHECKLIST and reporting guideline (optional).

Mandatory reporting items are marked with a red *.
In the textboxes, either copy & paste the relevant sections from your manuscript into this form - please include any quotes from your manuscript in QUOTATION MARKS,
or answer directly by providing additional information not in the manuscript, or elaborating on why the item was not relevant for this study.

YOUR ANSWERS WILL BE PUBLISHED AS A SUPPLEMENTARY FILE TO YOUR PUBLICATION IN JMIR AND ARE CONSIDERED PART OF YOUR PUBLICATION (IF ACCEPTED).
Please fill in these questions diligently. Information will not be copyedited, so please use proper spelling and grammar, use correct capitalization, and avoid abbreviations.

DO NOT FORGET TO SAVE AS PDF _AND_ CLICK THE SUBMIT BUTTON SO YOUR ANSWERS ARE IN OUR DATABASE !!!

Citation Suggestion (if you append the pdf as Appendix we suggest to cite this paper in the caption):
Eysenbach G, CONSORT-EHEALTH Group

CONSORT-EHEALTH: Improving and Standardizing Evaluation Reports of Web-based and Mobile Health Interventions
J Med Internet Res 2011;13(4):e126
URL: http://www.jmir.org/2011/4/e126/
doi: 10.2196/jmir.1923
PMID: 22209829

sabrinagiguere17@gmail.com Changer de compte

Non partagé

Brouillon enregistré

* Indique une question obligatoire

Your name *

First Last

Sabrina Giguère

Primary Affiliation (short), City, Country *

University of Toronto, Toronto, Canada

University of Montreal

Your e-mail address *

abc@gmail.com

sabrina.giguere.2@umontreal.ca

Title of your manuscript *

Provide the (draft) title of your manuscript.

Virtual Reality-Assisted Therapy Compared to Cognitive Behavioral Therapy for Patients with Treatment-Resistant Schizophrenia: A Randomized, Assessor-Blind Controlled Trial

Name of your App/Software/Intervention *

If there is a short and a long/alternate name, write the short name first and add the long name in brackets.

Virtual Reality-Assisted Therapy

Evaluated Version (if any)

e.g. "V1", "Release 2017-03-01", "Version 2.0.27913"

V1 Submitted 11-07-2026

Language(s) *

What language is the intervention/app in? If multiple languages are available, separate by comma (e.g. "English, French")

French

URL of your Intervention Website or App

e.g. a direct link to the mobile app on app in appstore (itunes, Google Play), or URL of the website. If the intervention is a DVD or hardware, you can also link to an Amazon page.

https://interventions-avatar.ca/

URL of an image/screenshot (optional)

Votre réponse

Accessibility *

Can an enduser access the intervention presently?

- ○ access is free and open
- ◉ access only for special usergroups, not open
- ○ access is open to everyone, but requires payment/subscription/in-app purchases
- ○ app/intervention no longer accessible
- ○ Autre :

Primary Medical Indication/Disease/Condition *

e.g. "Stress", "Diabetes", or define the target group in brackets after the condition, e.g. "Autism (Parents of children with)", "Alzheimers (Informal Caregivers of)"

"Individuals with schizophrenia or schizoaffect

Primary Outcomes measured in trial *

comma-separated list of primary outcomes reported in the trial

Auditory verbal hallucinations severity

Secondary/other outcomes

Are there any other outcomes the intervention is expected to affect?

Delusions
Symptoms of schizophrenia
Patients’ beliefs about their voices and related coping mechanisms
Acceptance-based attitudes and behaviors toward voice-hearing experiences
Self-esteem
Ability to understand, accept, and manage emotions effectively
The quality of life and psychosocial functioning

Recommended "Dose" *

What do the instructions for users say on how often the app should be used?

- ○ Approximately Daily
- ◉ Approximately Weekly
- ○ Approximately Monthly
- ○ Approximately Yearly
- ○ "as needed"
- ○ Autre :

Approx. Percentage of Users (starters) still using the app as recommended after 3 months *

- (selected) unknown / not evaluated
- 0-10%
- 11-20%
- 21-30%
- 31-40%
- 41-50%
- 51-60%
- 61-70%
- 71%-80%
- 81-90%
- 91-100%
- Autre :

Overall, was the app/intervention effective? *

- (selected) yes: all primary outcomes were significantly better in intervention group vs control
- partly: SOME primary outcomes were significantly better in intervention group vs control
- no statistically significant difference between control and intervention
- potentially harmful: control was significantly better than intervention in one or more outcomes
- inconclusive: more research is needed
- Autre :

Article Preparation Status/Stage *

At which stage in your article preparation are you currently (at the time you fill in this form)

- ○ not submitted yet - in early draft status
- ○ not submitted yet - in late draft status, just before submission
- ◉ submitted to a journal but not reviewed yet
- ○ submitted to a journal and after receiving initial reviewer comments
- ○ submitted to a journal and accepted, but not published yet
- ○ published
- ○ Autre :

Journal *

If you already know where you will submit this paper (or if it is already submitted), please provide the journal name (if it is not JMIR, provide the journal name under "other")

- ○ not submitted yet / unclear where I will submit this
- ○ Journal of Medical Internet Research (JMIR)
- ○ JMIR mHealth and UHealth
- ○ JMIR Serious Games
- ◉ JMIR Mental Health
- ○ JMIR Public Health
- ○ JMIR Formative Research
- ○ Other JMIR sister journal
- ○ Autre :

Is this a full powered effectiveness trial or a pilot/feasibility trial? *

- ○ Pilot/feasibility
- ◉ Fully powered

Manuscript tracking number *

If this is a JMIR submission, please provide the manuscript tracking number under "other" (The ms tracking number can be found in the submission acknowledgement email, or when you login as author in JMIR. If the paper is already published in JMIR, then the ms tracking number is the four-digit number at the end of the DOI, to be found at the bottom of each published article in JMIR)

- ○ no ms number (yet) / not (yet) submitted to / published in JMIR
- ◉ Autre : JMH ms#106791

TITLE AND ABSTRACT

1a) TITLE: Identification as a randomized trial in the title

1a) Does your paper address CONSORT item 1a? *

I.e does the title contain the phrase "Randomized Controlled Trial"? (if not, explain the reason under "other")

- ◉ yes
- ○ Autre :

1a-i) Identify the mode of delivery in the title

Identify the mode of delivery. Preferably use “web-based” and/or “mobile” and/or “electronic game” in the title. Avoid ambiguous terms like “online”, “virtual”, “interactive”. Use “Internet-based” only if Intervention includes non-web-based Internet components (e.g. email), use “computer-based” or “electronic” only if offline products are used. Use “virtual” only in the context of “virtual reality” (3-D worlds). Use “online” only in the context of “online support groups”. Complement or substitute product names with broader terms for the class of products (such as “mobile” or “smart phone” instead of “iphone”), especially if the application runs on different platforms.

	1	2	3	4	5	
subitem not at all important	○	○	○	○	◉	essential

Effacer la sélection

Does your paper address subitem 1a-i? *

Copy and paste relevant sections from manuscript title (include quotes in quotation marks "like this" to indicate direct quotes from your manuscript), or elaborate on this item by providing additional information not in the ms, or briefly explain why the item is not applicable/relevant for your study

"Virtual Reality-Assisted Therapy Compared to Cognitive Behavioral Therapy"

1a-ii) Non-web-based components or important co-interventions in title

Mention non-web-based components or important co-interventions in title, if any (e.g., “with telephone support”).

	1	2	3	4	5	
subitem not at all important	○	○	◉	○	○	essential

Effacer la sélection

Does your paper address subitem 1a-ii?

Copy and paste relevant sections from manuscript title (include quotes in quotation marks "like this" to indicate direct quotes from your manuscript), or elaborate on this item by providing additional information not in the ms, or briefly explain why the item is not applicable/relevant for your study

"Virtual Reality-Assisted Therapy Compared to Cognitive Behavioral Therapy"

1a-iii) Primary condition or target group in the title

Mention primary condition or target group in the title, if any (e.g., “for children with Type I Diabetes”) Example: A Web-based and Mobile Intervention with Telephone Support for Children with Type I Diabetes: Randomized Controlled Trial

	1	2	3	4	5	
subitem not at all important	○	○	○	○	◉	essential

Effacer la sélection

Does your paper address subitem 1a-iii? *

Copy and paste relevant sections from manuscript title (include quotes in quotation marks "like this" to indicate direct quotes from your manuscript), or elaborate on this item by providing additional information not in the ms, or briefly explain why the item is not applicable/relevant for your study

"Patients with Treatment-Resistant Schizophrenia"

1b) ABSTRACT: Structured summary of trial design, methods, results, and conclusions

NPT extension: Description of experimental treatment, comparator, care providers, centers, and blinding status.

1b-i) Key features/functionalities/components of the intervention and comparator in the METHODS section of the ABSTRACT

Mention key features/functionalities/components of the intervention and comparator in the abstract. If possible, also mention theories and principles used for designing the site. Keep in mind the needs of systematic reviewers and indexers by including important synonyms. (Note: Only report in the abstract what the main paper is reporting. If this information is missing from the main body of text, consider adding it)

	1	2	3	4	5	
subitem not at all important	○	○	○	◉	○	essential

Effacer la sélection

Does your paper address subitem 1b-i? *

Copy and paste relevant sections from the manuscript abstract (include quotes in quotation marks "like this" to indicate direct quotes from your manuscript), or elaborate on this item by providing additional information not in the ms, or briefly explain why the item is not applicable/relevant for your study

"VRT or CBT": Virtual reality-assisted therapy or cognitive behavioral therapy

1b-ii) Level of human involvement in the METHODS section of the ABSTRACT

Clarify the level of human involvement in the abstract, e.g., use phrases like “fully automated” vs. “therapist/nurse/care provider/physician-assisted” (mention number and expertise of providers involved, if any). (Note: Only report in the abstract what the main paper is reporting. If this information is missing from the main body of text, consider adding it)

	1	2	3	4	5	
subitem not at all important	○	○	○	◉	○	essential

Effacer la sélection

Does your paper address subitem 1b-ii?

Copy and paste relevant sections from the manuscript abstract (include quotes in quotation marks "like this" to indicate direct quotes from your manuscript), or elaborate on this item by providing additional information not in the ms, or briefly explain why the item is not applicable/relevant for your study

"Both nine-session therapies targeted maladaptive beliefs and relationships with voices and were delivered by therapists."

1b-iii) Open vs. closed, web-based (self-assessment) vs. face-to-face assessments in the METHODS section of the ABSTRACT

Mention how participants were recruited (online vs. offline), e.g., from an open access website or from a clinic or a closed online user group (closed usergroup trial), and clarify if this was a purely web-based trial, or there were face-to-face components (as part of the intervention or for assessment). Clearly say if outcomes were self-assessed through questionnaires (as common in web-based trials). Note: In traditional offline trials, an open trial (open-label trial) is a type of clinical trial in which both the researchers and participants know which treatment is being administered. To avoid confusion, use “blinded” or “unblinded” to indicated the level of blinding instead of “open”, as “open” in web-based trials usually refers to “open access” (i.e. participants can self-enrol). (Note: Only report in the abstract what the main paper is reporting. If this information is missing from the main body of text, consider adding it)

	1	2	3	4	5	
subitem not at all important	○	○	○	◉	○	essential

Effacer la sélection

Does your paper address subitem 1b-iii?

Copy and paste relevant sections from the manuscript abstract (include quotes in quotation marks "like this" to indicate direct quotes from your manuscript), or elaborate on this item by providing additional information not in the ms, or briefly explain why the item is not applicable/relevant for your study

"either referred by their healthcare team of self-refereed." "Psychotherapy sessions and assessments were conducted primarily in person, occasionally taking place via videoconference during the COVID-19 pandemic."

1b-iv) RESULTS section in abstract must contain use data

Report number of participants enrolled/assessed in each group, the use/uptake of the intervention (e.g., attrition/adherence metrics, use over time, number of logins etc.), in addition to primary/secondary outcomes. (Note: Only report in the abstract what the main paper is reporting. If this information is missing from the main body of text, consider adding it)

	1	2	3	4	5	
subitem not at all important	○	○	○	◉	○	essential

Effacer la sélection

Does your paper address subitem 1b-iv?

Copy and paste relevant sections from the manuscript abstract (include quotes in quotation marks "like this" to indicate direct quotes from your manuscript), or elaborate on this item by providing additional information not in the ms, or briefly explain why the item is not applicable/relevant for your study

"(VRT n=67; CBT n=69)"

1b-v) CONCLUSIONS/DISCUSSION in abstract for negative trials

Conclusions/Discussions in abstract for negative trials: Discuss the primary outcome - if the trial is negative (primary outcome not changed), and the intervention was not used, discuss whether negative results are attributable to lack of uptake and discuss reasons. (Note: Only report in the abstract what the main paper is reporting. If this information is missing from the main body of text, consider adding it)

	1	2	3	4	5	
subitem not at all important	○	○	○	◉	○	essential

Effacer la sélection

Does your paper address subitem 1b-v?

Copy and paste relevant sections from the manuscript abstract (include quotes in quotation marks "like this" to indicate direct quotes from your manuscript), or elaborate on this item by providing additional information not in the ms, or briefly explain why the item is not applicable/relevant for your study

"VRT outperformed a short course CBT in reducing persistent AVH in treatment-resistant schizophrenia. Results on secondary outcomes, such as the general psychotic symptomatology, were similar for both interventions. Overall, these findings support VRT's value as a personalized and clinically effective intervention."

INTRODUCTION

2a) In INTRODUCTION: Scientific background and explanation of rationale

2a-i) Problem and the type of system/solution

Describe the problem and the type of system/solution that is object of the study: intended as stand-alone intervention vs. incorporated in broader health care program? Intended for a particular patient population? Goals of the intervention, e.g., being more cost-effective to other interventions, replace or complement other solutions? (Note: Details about the intervention are provided in “Methods” under 5)

	1	2	3	4	5	
subitem not at all important	○	○	○	○	◉	essential

Effacer la sélection

Does your paper address subitem 2a-i? *

Copy and paste relevant sections from the manuscript (include quotes in quotation marks "like this" to indicate direct quotes from your manuscript), or elaborate on this item by providing additional information not in the ms, or briefly explain why the item is not applicable/relevant for your study

"To date, most randomized controlled trials (RCTs) have evaluated Avatar Therapy against minimally active or inactive control conditions, such as supportive counseling or treatment-as-usual [15, 18, 19, 21]. Consequently, whether it confers superiority over an established, evidence-based psychotherapy in reducing AVH severity remains an unresolved empirical question. The present trial addresses a critical gap in the literature as the first adequately powered assessor-blind RCT conducted exclusively in individuals with treatment-resistant schizophrenia and the first to compare VRT against CBT, the gold standard psychotherapeutic intervention for the treatment of psychosis."

2a-ii) Scientific background, rationale: What is known about the (type of) system

Scientific background, rationale: What is known about the (type of) system that is the object of the study (be sure to discuss the use of similar systems for other conditions/diagnoses, if appropiate), motivation for the study, i.e. what are the reasons for and what is the context for this specific study, from which stakeholder viewpoint is the study performed, potential impact of findings [2]. Briefly justify the choice of the comparator.

	1	2	3	4	5	
subitem not at all important	○	○	○	○	◉	essential

Effacer la sélection

Does your paper address subitem 2a-ii? *

Copy and paste relevant sections from the manuscript (include quotes in quotation marks "like this" to indicate direct quotes from your manuscript), or elaborate on this item by providing additional information not in the ms, or briefly explain why the item is not applicable/relevant for your study

"Auditory verbal hallucinations (AVH) are highly prevalent and clinically distressing residual symptoms, hence why psychotherapeutic frameworks often target them [8, 9]. Notably, using a structured, goal-oriented approach aimed at reducing distress and anxiety, cognitive behavioral therapy (CBT) demonstrated low-to-moderate short-term effects on AVH severity and overall positive symptoms [3, 10]. Nevertheless, its efficacy appears to be limited over time, highlighting the need to develop alternative non-pharmacological treatment modalities [11, 12].
Novel psychological approaches recently emerged, including relational therapies grounded in an interpersonal conceptualization of voice-hearing, such as Avatar Therapy [13, 14]. Through real-time digital animation and voice modulation, patients are invited to engage with a visual representation of a distressing voice, animated and controlled by a therapist. Initially developed in the United Kingdom, it has subsequently been adapted worldwide, including in Canada, through the integration of immersive three-dimensional virtual reality (VR) [15-21]. The outcomes of the Virtual Reality-assisted Therapy (VRT) have consistently demonstrated promising effects on AVH, affective symptoms, and beliefs about voices [9, 16, 17, 20, 21]."

2b) In INTRODUCTION: Specific objectives or hypotheses

Does your paper address CONSORT subitem 2b? *

Copy and paste relevant sections from the manuscript (include quotes in quotation marks "like this" to indicate direct quotes from your manuscript), or elaborate on this item by providing additional information not in the ms, or briefly explain why the item is not applicable/relevant for your study

"Hence, the primary objective of this trial was to determine whether VRT is superior to a short course CBT adapted to serve as an active comparator (same duration and similar therapeutic targets) in reducing the impact of AVH in treatment-resistant schizophrenia. Secondary objectives included the evaluation of treatment effects on other positive psychotic symptoms, general psychopathology, beliefs about and responses to voices, emotion regulation, and self-esteem. Consistent with prior clinical evidence, we hypothesized that VRT would demonstrate superiority over CBT in reducing AVH severity and that participants in the VRT group would exhibit greater improvements in positive psychotic symptoms and emotional regulation."

METHODS

3a) Description of trial design (such as parallel, factorial) including allocation ratio

Does your paper address CONSORT subitem 3a? *

Copy and paste relevant sections from the manuscript (include quotes in quotation marks "like this" to indicate direct quotes from your manuscript), or elaborate on this item by providing additional information not in the ms, or briefly explain why the item is not applicable/relevant for your study

"The present study consisted of a randomized, controlled, assessor-blind, parallel-group clinical trial comparing two interventions: VRT and a short course CBT protocol focused on auditory verbal hallucinations (AVH)." "After completion of the baseline assessment, participants were randomly assigned in a 1:1 ratio to VRT or CBT via an independent, secure, Internet-based service sequence generation process (https://www.sealedenvelope.com), using randomly varying block sizes of two, four, and six, stratified by gender and baseline clozapine treatment. "

3b) Important changes to methods after trial commencement (such as eligibility criteria), with reasons

Does your paper address CONSORT subitem 3b? *

Copy and paste relevant sections from the manuscript (include quotes in quotation marks "like this" to indicate direct quotes from your manuscript), or elaborate on this item by providing additional information not in the ms, or briefly explain why the item is not applicable/relevant for your study

There were no important changes to the trial methods after trial commencement.

3b-i) Bug fixes, Downtimes, Content Changes

Bug fixes, Downtimes, Content Changes: ehealth systems are often dynamic systems. A description of changes to methods therefore also includes important changes made on the intervention or comparator during the trial (e.g., major bug fixes or changes in the functionality or content) (5-iii) and other "unexpected events" that may have influenced study design such as staff changes, system failures/downtimes, etc. [2].

	1	2	3	4	5	
subitem not at all important	○	○	○	●	○	essential

Effacer la sélection

Does your paper address subitem 3b-i?

Copy and paste relevant sections from the manuscript (include quotes in quotation marks "like this" to indicate direct quotes from your manuscript), or elaborate on this item by providing additional information not in the ms, or briefly explain why the item is not applicable/relevant for your study

The COVID-19 pandemic led to occasional delivery of CBT sessions and some study assessments via videoconference. No other unexpected events influenced the study design or conduct. "Although it was initially intended to be delivered in-person like VRT, CBT was occasionally delivered via videoconference during the COVID-19 pandemic." "In addition, the COVID-19 pandemic did not increase the number of participants with protocol deviations, but during this period, the longest delays between assessments were observed in both study arms. To minimize these delays, some evaluations were conducted remotely via videoconference. "

4a) Eligibility criteria for participants

Does your paper address CONSORT subitem 4a? *

Copy and paste relevant sections from the manuscript (include quotes in quotation marks "like this" to indicate direct quotes from your manuscript), or elaborate on this item by providing additional information not in the ms, or briefly explain why the item is not applicable/relevant for your study

"Participants were eligible if they were 18 years of age or older, diagnosed with either schizophrenia or schizoaffective disorder with persistent AVH, non-responsive to two or more antipsychotic trials, each lasting more than 6 weeks at a therapeutic dosage equivalent to at least 600mg of chlorpromazine daily, and on stable doses of medication during the two months before study enrollment with a good adherence (defined as 80% or more of the doses being taken). Participants were excluded if they presented a neurological disorder, an unstable and serious physical illness, a substance use disorder in the past year, had received CBT for psychosis in the past year, lacked the capacity to consent, or were currently experiencing an acute psychotic episode. These criteria were similar to those of other trials in the field of treatment-resistant schizophrenia [21-23]. With the participant’s consent, attempts were made to contact each participant's treating psychiatrist to confirm eligibility, inform them of their participation in the clinical trial, and ensure that no changes in psychiatric medication were planned in the near future. Moreover, participants could be excluded during therapy if their mental state deteriorated and prevented them from continuing the treatment."

4a-i) Computer / Internet literacy

Computer / Internet literacy is often an implicit “de facto” eligibility criterion - this should be explicitly clarified.

	1	2	3	4	5	
subitem not at all important	◉	○	○	○	○	essential

Effacer la sélection

Does your paper address subitem 4a-i?

Copy and paste relevant sections from the manuscript (include quotes in quotation marks "like this" to indicate direct quotes from your manuscript), or elaborate on this item by providing additional information not in the ms, or briefly explain why the item is not applicable/relevant for your study

Participation did not require computer or internet literacy.

4a-ii) Open vs. closed, web-based vs. face-to-face assessments:

Open vs. closed, web-based vs. face-to-face assessments: Mention how participants were recruited (online vs. offline), e.g., from an open access website or from a clinic, and clarify if this was a purely web-based trial, or there were face-to-face components (as part of the intervention or for assessment), i.e., to what degree got the study team to know the participant. In online-only trials, clarify if participants were quasi-anonymous and whether having multiple identities was possible or whether technical or logistical measures (e.g., cookies, email confirmation, phone calls) were used to detect/prevent these.

	1	2	3	4	5	
subitem not at all important	○	○	○	◉	○	essential

Effacer la sélection

Does your paper address subitem 4a-ii? *

Copy and paste relevant sections from the manuscript (include quotes in quotation marks "like this" to indicate direct quotes from your manuscript), or elaborate on this item by providing additional information not in the ms, or briefly explain why the item is not applicable/relevant for your study

"Assessments were conducted primarily in person, occasionally taking place via videoconference during the COVID-19 pandemic."

4a-iii) Information giving during recruitment

Information given during recruitment. Specify how participants were briefed for recruitment and in the informed consent procedures (e.g., publish the informed consent documentation as appendix, see also item X26), as this information may have an effect on user self-selection, user expectation and may also bias results.

	1	2	3	4	5	
subitem not at all important	○	○	○	○	◉	essential

Effacer la sélection

Does your paper address subitem 4a-iii?

Copy and paste relevant sections from the manuscript (include quotes in quotation marks "like this" to indicate direct quotes from your manuscript), or elaborate on this item by providing additional information not in the ms, or briefly explain why the item is not applicable/relevant for your study

"Recruitment occurred between April 2019 and June 2025. Participants were either referred by their healthcare team or self-referred. Active efforts were made to inform healthcare providers about the project in four psychiatric institutions across the province, including the major psychiatric institutions on the island of Montreal, as well as in other urban and suburban areas, and online via videoconference. Presentations were also offered to patients and their loved ones through community organizations such as the Société Québécoise de la Schizophrénie and the Association des parents et amis de la personne atteinte de maladie mentale. Information about the project was available on a dedicated website, which included a self-referral form and contact information (phone number and email address) for more details on study eligibility. However, participants could come from anywhere in the province."

"The trial was conducted in accordance with the Declaration of Helsinki. It was approved by the institutional ethical committee of the Centre intégré universitaire de santé et de services sociaux (CIUSSS) de l'Est-de-l'Île-de-Montréal (project number: MP-12-2019-1726) in April 2019. Prior to their inclusion in the study, the Information and Consent Form was carefully presented and explained to potential participants. The paper form was also provided, along with time and space to make an informed decision. All questions were answered as needed, whether during the presentation or later by phone. Capacity to consent was also assessed; for example, participants were asked to summarise different sections of the form. If they agreed to participate and had capacity, they were allowed to provide written informed consent, and basic information was collected during the same visit, including sociodemographic data, contact information, and whether they were currently using clozapine (for randomization purposes)."

4b) Settings and locations where the data were collected

Does your paper address CONSORT subitem 4b? *

Copy and paste relevant sections from the manuscript (include quotes in quotation marks "like this" to indicate direct quotes from your manuscript), or elaborate on this item by providing additional information not in the ms, or briefly explain why the item is not applicable/relevant for your study

"Finally, assessment data from paper questionnaires were entered into a secure database by blinded research assistants. Data entry was performed twice independently to minimize transcription errors."

4b-i) Report if outcomes were (self-)assessed through online questionnaires

Clearly report if outcomes were (self-)assessed through online questionnaires (as common in web-based trials) or otherwise.

	1	2	3	4	5	
subitem not at all important	○	○	○	●	○	essential

Effacer la sélection

Does your paper address subitem 4b-i? *

Copy and paste relevant sections from the manuscript (include quotes in quotation marks "like this" to indicate direct quotes from your manuscript), or elaborate on this item by providing additional information not in the ms, or briefly explain why the item is not applicable/relevant for your study

"Standardized assessments were performed one week before the first therapy session (pre-therapy), one week after the last session (post-therapy), and three months after the last therapy session (3-month follow-up) by a research assistant and nurses. Assessments were conducted primarily in person, occasionally taking place via videoconference during the COVID-19 pandemic."

4b-ii) Report how institutional affiliations are displayed

Report how institutional affiliations are displayed to potential participants [on ehealth media], as affiliations with prestigious hospitals or universities may affect volunteer rates, use, and reactions with regards to an intervention.(Not a required item – describe only if this may bias results)

	1	2	3	4	5	
subitem not at all important	○	○	○	○	◉	essential

Effacer la sélection

Does your paper address subitem 4b-ii?

Copy and paste relevant sections from the manuscript (include quotes in quotation marks "like this" to indicate direct quotes from your manuscript), or elaborate on this item by providing additional information not in the ms, or briefly explain why the item is not applicable/relevant for your study

The institutional affiliations of the investigators are provided in the Information and Consent Form.

5) The interventions for each group with sufficient details to allow replication, including how and when they were actually administered

5-i) Mention names, credential, affiliations of the developers, sponsors, and owners

Mention names, credential, affiliations of the developers, sponsors, and owners [6] (if authors/evaluators are owners or developer of the software, this needs to be declared in a “Conflict of interest” section or mentioned elsewhere in the manuscript).

	1	2	3	4	5	
subitem not at all important	○	○	○	◉	○	essential

Effacer la sélection

Does your paper address subitem 5-i?

Copy and paste relevant sections from the manuscript (include quotes in quotation marks "like this" to indicate direct quotes from your manuscript), or elaborate on this item by providing additional information not in the ms, or briefly explain why the item is not applicable/relevant for your study

"The VR platform used was developed by OVA."
"The VR platform used in this study was developed by OVA and is owned by AD Psychiatric Services."

5-ii) Describe the history/development process

Describe the history/development process of the application and previous formative evaluations (e.g., focus groups, usability testing), as these will have an impact on adoption/use rates and help with interpreting results.

	1	2	3	4	5	
subitem not at all important	○	○	○	○	●	essential

Effacer la sélection

Does your paper address subitem 5-ii?

Copy and paste relevant sections from the manuscript (include quotes in quotation marks "like this" to indicate direct quotes from your manuscript), or elaborate on this item by providing additional information not in the ms, or briefly explain why the item is not applicable/relevant for your study

"VRT is a psychological intervention designed to treat individuals with treatment-resistant schizophrenia and persistent AVH by engaging them with a digital representation ("avatar") of their most distressing hallucination. It was initially developed in the United Kingdom [19] and was adapted using virtual reality by Dr. Alexandre Dumais. Following the first pilot trial conducted by his research team, it was determined that nine therapy sessions were optimal for maximizing outcomes and adequately consolidating learning [17]. Therefore, this therapy consists of nine weekly 1-hour sessions."

5-iii) Revisions and updating

Revisions and updating. Clearly mention the date and/or version number of the application/intervention (and comparator, if applicable) evaluated, or describe whether the intervention underwent major changes during the evaluation process, or whether the development and/or content was "frozen" during the trial. Describe dynamic components such as news feeds or changing content which may have an impact on the replicability of the intervention (for unexpected events see item 3b).

	1	2	3	4	5	
subitem not at all important	○	○	○	○	◉	essential

Effacer la sélection

Does your paper address subitem 5-iii?

Copy and paste relevant sections from the manuscript (include quotes in quotation marks "like this" to indicate direct quotes from your manuscript), or elaborate on this item by providing additional information not in the ms, or briefly explain why the item is not applicable/relevant for your study

There were no major modifications to the intervention during the trial.

5-iv) Quality assurance methods

Provide information on quality assurance methods to ensure accuracy and quality of information provided [1], if applicable.

	1	2	3	4	5	
subitem not at all important	○	○	○	○	◉	essential

Effacer la sélection

Does your paper address subitem 5-iv?

Copy and paste relevant sections from the manuscript (include quotes in quotation marks "like this" to indicate direct quotes from your manuscript), or elaborate on this item by providing additional information not in the ms, or briefly explain why the item is not applicable/relevant for your study

"Surveillance, Protocol Deviations, and Treatment Fidelity
Protocol deviations and undesirable treatment effects were compiled, analyzed, and presented by a graduate student (MB). Five categories of deviation were documented, including assessment timing, evaluator blinding, changes in antipsychotic medications, and treatment fidelity. The study was then overseen regularly by a steering committee that included the involved researchers, clinicians, a graduate student (MB), the president of the ethics committee, a biostatistician (CEG), and one patient-partner. After each of these meetings, the committee had to vote to continue the study, and the vote had to be unanimous to allow continuation. Moreover, the committee's mandate was to recommend future adjustments to the study, notably to minimize and manage protocol deviations. If serious adverse events had occurred, a protocol was in place to ensure they would have been handled seriously, including careful revision of the event by an independent committee to determine whether it was linked to therapy and what could have been done to prevent it, and the writing of a notice to the study Ethics committee.
Treatment fidelity was evaluated across 120 randomly selected sessions from both conditions, comprising 60 sessions from the cognitive-behavioral therapy condition and 60 sessions from the VRT condition. Adherence to the respective treatment manuals was assessed using structured fidelity checklists developed specifically for each intervention."

5-v) Ensure replicability by publishing the source code, and/or providing screenshots/screen-capture video, and/or providing flowcharts of the algorithms used

Ensure replicability by publishing the source code, and/or providing screenshots/screen-capture video, and/or providing flowcharts of the algorithms used. Replicability (i.e., other researchers should in principle be able to replicate the study) is a hallmark of scientific reporting.

	1	2	3	4	5	
subitem not at all important	○	○	◉	○	○	essential

Effacer la sélection

Does your paper address subitem 5-v?

Copy and paste relevant sections from the manuscript (include quotes in quotation marks "like this" to indicate direct quotes from your manuscript), or elaborate on this item by providing additional information not in the ms, or briefly explain why the item is not applicable/relevant for your study

We do not hold the source code. The application was designed by a company.

5-vi) Digital preservation

Digital preservation: Provide the URL of the application, but as the intervention is likely to change or disappear over the course of the years; also make sure the intervention is archived (Internet Archive, webcitation.org, and/or publishing the source code or screenshots/videos alongside the article). As pages behind login screens cannot be archived, consider creating demo pages which are accessible without login.

	1	2	3	4	5	
subitem not at all important	◉	○	○	○	○	essential

Effacer la sélection

Does your paper address subitem 5-vi?

Copy and paste relevant sections from the manuscript (include quotes in quotation marks "like this" to indicate direct quotes from your manuscript), or elaborate on this item by providing additional information not in the ms, or briefly explain why the item is not applicable/relevant for your study

Not available in open access.

5-vii) Access

Access: Describe how participants accessed the application, in what setting/context, if they had to pay (or were paid) or not, whether they had to be a member of specific group. If known, describe how participants obtained "access to the platform and Internet" [1]. To ensure access for editors/reviewers/readers, consider to provide a "backdoor" login account or demo mode for reviewers/readers to explore the application (also important for archiving purposes, see vi).

	1	2	3	4	5	
subitem not at all important	○	○	○	○	◉	essential

Effacer la sélection

Does your paper address subitem 5-vii? *

Copy and paste relevant sections from the manuscript (include quotes in quotation marks "like this" to indicate direct quotes from your manuscript), or elaborate on this item by providing additional information not in the ms, or briefly explain why the item is not applicable/relevant for your study

"The therapies were delivered at the Institut universitaire en santé mentale de Montréal located in Montreal, Canada."

5-viii) Mode of delivery, features/functionalities/components of the intervention and comparator, and the theoretical framework

Describe mode of delivery, features/functionalities/components of the intervention and comparator, and the theoretical framework [6] used to design them (instructional strategy [1], behaviour change techniques, persuasive features, etc., see e.g., [7, 8] for terminology). This includes an in-depth description of the content (including where it is coming from and who developed it) [1]," whether [and how] it is tailored to individual circumstances and allows users to track their progress and receive feedback" [6]. This also includes a description of communication delivery channels and – if computer-mediated communication is a component – whether communication was synchronous or asynchronous [6]. It also includes information on presentation strategies [1], including page design principles, average amount of text on pages, presence of hyperlinks to other resources, etc. [1].

	1	2	3	4	5	
subitem not at all important	○	○	○	○	◉	essential

Effacer la sélection

Does your paper address subitem 5-viii? *

Copy and paste relevant sections from the manuscript (include quotes in quotation marks "like this" to indicate direct quotes from your manuscript), or elaborate on this item by providing additional information not in the ms, or briefly explain why the item is not applicable/relevant for your study

"Therapies
Virtual Reality-Assisted Therapy
VRT is a psychological intervention designed to treat individuals with treatment-resistant schizophrenia and persistent AVH by engaging them with a digital representation (“avatar”) of their most distressing hallucination. It was initially developed in the United Kingdom [19] and was adapted using virtual reality by Dr. Alexandre Dumais. Following the first pilot trial conducted by his research team, it was determined that nine therapy sessions were optimal for maximizing outcomes and adequately consolidating learning [17]. Therefore, this therapy consists of nine weekly 1-hour sessions.
The first session consisted of a case formulation focusing on the content and context surrounding the AVH, as well as the participants’ coping strategies. In doing so, the participants were invited to identify their most distressing voice and to imagine what it would look like. Then, participants were asked to create an avatar representing that voice. This step was conducted in a virtual reality environment using an Oculus Rift head-mounted display. With the therapist’s support, participants customized their avatar’s physical appearance (e.g., facial features, eyes, hair, skin color, haircut) and voice characteristics (e.g., pitch, volume) to closely match the voice they were hearing. This was done using a custom Unity 3D game engine, with unique avatars generated via the Morph3D Character System. Finally, participants were asked to write down the content of their hallucinations on a typical day before the next session.
During subsequent sessions, participants were invited to engage in dialogue with their avatar in virtual reality, which the therapist animated in real time. Playing the role of the avatar, the therapist spoke into a microphone while their voice was transformed in real time using the Roland AIRA VT-3 voice transformer. Lip synchronization between the therapist’s speech and the Avatar was achieved with the SALSA with Random Eyes Unity 3D extension. Facial expressions could also be modulated by the therapist; they were programmed to convey emotions using the Facial Action Coding System. The virtual environment consisted of a three-dimensional avatar standing in a neutral single-coloured cubic room, viewed from a first-person perspective. The participants and their therapist were separated by a one-way mirror, allowing the therapist to observe the participant during the session. Please refer to Figure S1 (Multimedia Appendix) for a visual of the setting in which the immersion took place.
Each therapeutic session (session 2 and onward) consisted of three components, each lasting approximately 5 to 20 minutes:
1. Pre-immersion: review of the previous week, review of the assignment (when applicable), and establishment of objectives for the current therapy session.
2. VR Immersion: the participants engage in a dialogue with their avatars. In doing so, they are invited to experiment with different coping strategies.
3. Post-immersion: following immersion, the participant’s experience was debriefed, focusing specifically on the emotions that arose during the session. When applicable, assignments were then given for the upcoming week.
During the second and third sessions, participants were exposed to an avatar that faithfully reproduced their hallucinatory experience. For this purpose, the therapist facilitated a dialogue between the participant and the avatar using the sentences the participant had previously been asked to record, faithfully capturing the content. In return, participants were encouraged to respond to the avatar using their usual coping strategies, as well as new ones suggested by the therapist, to promote self-assertiveness. Patients were therefore provided with a safe, confidential therapeutic space to express their emotions, opinions, and

demands. In doing so, participants were supported in challenging the perceived omnipotence of the voice. Starting in the third session, the avatar started to open up and consider what the participant was telling them.
During the fourth session, the avatar became less confrontational/threatening and began to make attempts to reconcile with the participant, namely by encouraging the participant to verbalize their emotions and demands. It could notably support the participant and help them in suggesting new interpretations related to their hallucinatory experiences; for example, it would suggest that there might be a link between weak self-esteem and derogatory voice content. At the end of this session, participants were required to ask a close relative or friend about their qualities and list those for the next session.
For the fifth session, the focus formally shifted to self-esteem, which was reinforced by encouraging participants to reflect on their personal qualities. Using the list provided by the participants, they were encouraged to incorporate these positive attributes into the dialogue with their avatar, using self-worth to invalidate deprecating comments.
In the final consolidation sessions (sixth to ninth), participants were encouraged to apply what they had learned in the experiential sessions. The aim was to highlight the participants' progress, promote relapse prevention, and support them in accepting and living with the residual voices. Participants were also guided in formulating aspirations for their future relationship with the voices as well as for moving forward with their lives.
Over the course of therapy, the therapist gradually modified the avatar's speech and tone to align with the participant's growing sense of empowerment, transforming the avatar from abusive to more helpful and supportive. Additionally, new suggestions regarding the participants' beliefs about the voices and their origins were introduced. During the immersive sessions, the therapist could also pause at any point, make the avatar disappear, and intervene as themselves to guide, encourage, or assess the participant's anxiety/fear levels.
Recordings of the immersive portion of each session were made available to participants during therapy, and they were strongly encouraged to listen to them between sessions.
Short Course Cognitive Behavioral Therapy for Auditory Verbal Hallucinations
The CBT therapy manual was developed collaboratively by one of the lead researchers, a CBT expert, and two psychologists with established proficiency and extensive clinical experience in delivering CBT. The manual was specifically adapted to serve as an active comparator to VRT, most notably by targeting the same primary outcome and adhering to an equivalent short course treatment format comprising 9 weekly, 1-hour sessions [10, 24].
The first therapy session was devoted to case formulation, focusing on exploring the participant's voice content, contextual factors, and strategies for managing AVH. The second session focused on psychoeducation and the normalization of hallucinatory experiences, including the use of well-known public figures who have reported similar phenomena, and the presentation of the vulnerability-stress model with concrete examples. AVH was also described and characterized, with particular attention to identifying triggers, contexts, and modulators associated with voice-hearing episodes. The third session focused on presenting the cognitive model of AVH, illustrated using a patient-specific example. Together with the patient, triggers were identified, followed by the anxiety-provoking thoughts and associated beliefs that ensued. The fourth session focused on learning about various attributional mechanisms. Through situational exercises and patient-generated examples, participants were encouraged to recognize that multiple interpretations can be drawn from the same event, thereby fostering greater cognitive flexibility. The fifth session focused on coping mechanisms. The therapist introduced multiple adaptation strategies (e.g., behavioral distraction, cognitive distraction, socialization) to help patients manage voice hearing experiences. Patients were encouraged

socialization) to help patients manage voice-hearing experiences. Patients were encouraged to reflect on which strategies they had attempted and to evaluate whether these strategies were effective in reducing distress. The sixth session focused on modifying beliefs and formulating alternative explanations. Using Socratic questioning, evidence evaluation, and behavioral experiments, the therapist supported the patient in generating more plausible alternative explanations for their AVH. Patients were encouraged to assess the likelihood of each interpretation through structured exercises, thereby testing and refining their beliefs. The seventh and eighth sessions focused on mindfulness-based exercises grounded in sensory awareness. Participants were instructed not to attempt to suppress the voices, but rather to observe and listen to them without acting on them or judging them. Mindfulness practice was encouraged during actual voice-hearing episodes occurring during therapy or through guided recall of situations in which voices had been present. The final session focused on consolidating the skills and coping strategies acquired throughout therapy. A collaborative relapse prevention plan was developed with the participant, outlining individualized strategies for early identification and management of exacerbations. Throughout therapy, various questionnaires and exercises were incorporated to facilitate learning. Moreover, participants were asked to complete homework between sessions, including required readings and a voice journal. This voice journal initially included when the voice occurred, the context, the intensity, and the associated emotions. As therapy progressed, participants were also invited to apply coping strategies and document their impact in this journal.
Although it was initially intended to be delivered in-person like VRT, CBT was occasionally delivered via videoconference during the COVID-19 pandemic. Although data is still limited for schizophrenia, videoconference-delivered individual CBT is generally considered to be as effective as in-person one-on-one CBT for most psychiatric conditions, with recent meta-analyses showing non-inferior outcomes between the two formats [25-27]."

5-ix) Describe use parameters

Describe use parameters (e.g., intended “doses” and optimal timing for use). Clarify what instructions or recommendations were given to the user, e.g., regarding timing, frequency, heaviness of use, if any, or was the intervention used ad libitum.

	1	2	3	4	5	
subitem not at all important	○	○	○	○	◉	essential

Effacer la sélection

Does your paper address subitem 5-ix?

Copy and paste relevant sections from the manuscript (include quotes in quotation marks "like this" to indicate direct quotes from your manuscript), or elaborate on this item by providing additional information not in the ms, or briefly explain why the item is not applicable/relevant for your study

VRT: "Therefore, this therapy consists of nine weekly 1-hour sessions."
CBT: "treatment format comprising 9 weekly, 1-hour sessions"

5-x) Clarify the level of human involvement

Clarify the level of human involvement (care providers or health professionals, also technical assistance) in the e-intervention or as co-intervention (detail number and expertise of professionals involved, if any, as well as “type of assistance offered, the timing and frequency of the support, how it is initiated, and the medium by which the assistance is delivered”. It may be necessary to distinguish between the level of human involvement required for the trial, and the level of human involvement required for a routine application outside of a RCT setting (discuss under item 21 – generalizability).

	1	2	3	4	5	
subitem not at all important	○	○	○	○	●	essential

Effacer la sélection

Does your paper address subitem 5-x?

Copy and paste relevant sections from the manuscript (include quotes in quotation marks "like this" to indicate direct quotes from your manuscript), or elaborate on this item by providing additional information not in the ms, or briefly explain why the item is not applicable/relevant for your study

"The therapy team comprised three psychologists, one psychiatrist with expertise in VRT, and two psychiatry residents under the supervision of the VRT expert. All therapists had prior clinical experience treating psychotic disorders (mean years of experience = 12.75, range = 1-20), and therapist profiles, including professional background and years of experience, were balanced across the two treatment arms."

5-xi) Report any prompts/reminders used

Report any prompts/reminders used: Clarify if there were prompts (letters, emails, phone calls, SMS) to use the application, what triggered them, frequency etc. It may be necessary to distinguish between the level of prompts/reminders required for the trial, and the level of prompts/reminders for a routine application outside of a RCT setting (discuss under item 21 – generalizability).

	1	2	3	4	5	
subitem not at all important	○	○	○	◉	○	essential

Effacer la sélection

Does your paper address subitem 5-xi? *

Copy and paste relevant sections from the manuscript (include quotes in quotation marks "like this" to indicate direct quotes from your manuscript), or elaborate on this item by providing additional information not in the ms, or briefly explain why the item is not applicable/relevant for your study

"In addition, the psychiatric nurse made a reminder phone call the day before each

5-xii) Describe any co-interventions (incl. training/support)

Describe any co-interventions (incl. training/support): Clearly state any interventions that are provided in addition to the targeted eHealth intervention, as ehealth intervention may not be designed as stand-alone intervention. This includes training sessions and support [1]. It may be necessary to distinguish between the level of training required for the trial, and the level of training for a routine application outside of a RCT setting (discuss under item 21 – generalizability.

	1	2	3	4	5	
subitem not at all important	○	○	○	◉	○	essential

Effacer la sélection

Does your paper address subitem 5-xii? *

Copy and paste relevant sections from the manuscript (include quotes in quotation marks "like this" to indicate direct quotes from your manuscript), or elaborate on this item by providing additional information not in the ms, or briefly explain why the item is not applicable/relevant for your study

"Training procedures encompassed direct observation of live therapy sessions, review of the therapy manual, and systematic listening to audio recordings of therapy sessions. Furthermore, each therapist's initial cases were conducted under the direct supervision of either the VRT expert or the CBT expert, the investigators responsible for developing and delivering the respective interventions in the pilot randomized trial [16]. Psychiatry residents additionally received weekly individual psychotherapy supervision from the VRT expert throughout the entire duration of their therapy delivery."

However, participants did not require any training or technical support to participate in the psychotherapies, as the interventions were therapist-delivered rather than stand-alone.

6a) Completely defined pre-specified primary and secondary outcome measures, including how and when they were assessed

Does your paper address CONSORT subitem 6a? *

Copy and paste relevant sections from the manuscript (include quotes in quotation marks "like this" to indicate direct quotes from your manuscript), or elaborate on this item by providing additional information not in the ms, or briefly explain why the item is not applicable/relevant for your study

"Standardized assessments were performed one week before the first therapy session (pre-therapy), one week after the last session (post-therapy), and three months after the last therapy session (3-month follow-up) by a research assistant and nurses. Assessments were conducted primarily in person, occasionally taking place via videoconference during the COVID-19 pandemic.
First, sociodemographic data (e.g., age, gender, ethnicity, education level, revenue) were collected prior to the first formal assessment. This visit generally occurred approximately one or two weeks prior to the first clinical assessment.
Then, the first standardized assessment was conducted in two visits, generally one or two weeks prior to the first therapy session (7 days). During the first part of the pre-therapy evaluation, the diagnosis of schizophrenia or schizoaffective disorder (as per DSM-5 criteria) was confirmed using the Structured Clinical Interview for the Diagnostic and Statistical Manual of Mental Disorders, Fifth Edition [28]. During the same visit, information was also collected on participants' age at first contact with psychiatry and diagnoses.
The second part of the evaluation was blinded and included all quantitative questionnaires and scales assessing the primary and secondary outcomes of the study; it was identical to those conducted post-therapy and at the 3-month follow-up. This quantitative evaluation included measures of the severity of AVH and delusions, general schizophrenia symptoms, beliefs about the voices, acceptance-based attitudes and behaviors toward voices, self-esteem, emotion regulation, and quality of life. The predetermined primary outcome of the randomized controlled trial was the overall severity of AVH, assessed using the total score of the Auditory Hallucinations subscale of the Psychotic Symptoms Rating Scale (PSYRATS-AH) at post-treatment and 3-month follow-up. The PSYRATS-AH is a well-validated, dimensional, semi-structured, assessor-rated clinical interview comprising 11 items that quantify multiple phenomenological dimensions of AVH, including frequency, loudness, voice-associated distress, and attribution (beliefs about the origin) [29]. This clinical tool has shown excellent interrater reliability (ICC=0.9) and good validity [29]. Of note, the PSYRATS-AH subscale was amended so that participants who no longer experienced hallucinations at the time of follow-up were assigned a total score of 0 for inclusion in the analyses.
Secondary outcomes were also assessed at post-treatment and at the 3-month follow-up. Delusions were assessed with the Delusions subscale of the Psychotic Symptoms Rating Scale (PSYRATS-D), which includes measures about the frequency of delusions and delusion-associated distress [29]. Symptoms of schizophrenia were evaluated with the Positive and Negative Syndrome Scale (PANSS) [30]. This scale was separated into five symptom clusters using its demonstrated factorial structure: positive, negative, disorganization, depression-anxiety, and excitement [31]. Patients' beliefs about their voices and related coping mechanisms were measured with the Beliefs About Voices Questionnaire-Revised (BAVQ-R), which includes three subscales assessing beliefs (i.e., malevolence, benevolence, omnipotence) and two subscales assessing the emotional and behavioral components of AVH (i.e., resistance, engagement) [32]. Similar to the PSYRATS, participants who no longer experienced AVH at follow-up were assigned a BAVQ-R total score of 0. Acceptance-based attitudes and behaviors toward voice-hearing experiences were assessed with the Voices Acceptance and Action Scale (VAAS) [33]. The self-esteem was assessed using the Rosenberg Self-Esteem Scale (RSES), and the ability to understand, accept, and manage emotions effectively was assessed using the Difficulties in Emotion Regulation Scale (DERS-18) [34, 35]. The quality of life and psychosocial functioning were assessed using the self-reported Quality of Life Enjoyment and Satisfaction Questionnaire Short Form (QLES-Q-SF) [36]. Additional details

Short Form (QLES-Q-SF) [36]. Additional details on all these tools, including their psychometric properties, are available in Table S1 (Multimedia Appendix).
All post-randomization evaluations were conducted by trained and experienced research professionals under blinded conditions. Assessors received training through interview videos and reading materials, with particular emphasis on instruments that require clinical judgment. To ensure interrater reliability, evaluators were trained together using standardized videotapes and consensus ratings. For each assessor, the first few assessments were conducted under the supervision of a lead researcher (A.D.), who provided constructive feedback. Regular team meetings were held to ensure good inter-rater reliability. To improve consistency, all quantitative assessments of a given participant were conducted by the same blinded evaluator.
The Negative Effects Questionnaire, an instrument designed to assess adverse and unwanted effects of psychological treatments, was administered [37]. It includes six domains: symptoms, quality of the treatment, dependency on the treatment or therapist, stigma (i.e., being perceived negatively by others because of undergoing treatment), hopelessness, and lowered self-esteem [37, 38]. Because of the particular risk of unblinding associated with this questionnaire, it was administered by an unblinded psychiatric nurse after the therapy.
Finally, assessment data from paper questionnaires were entered into a secure database by blinded research assistants. Data entry was performed twice independently to minimize transcription errors."

6a-i) Online questionnaires: describe if they were validated for online use and apply CHERRIES items to describe how the questionnaires were designed/deployed

If outcomes were obtained through online questionnaires, describe if they were validated for online use and apply CHERRIES items to describe how the questionnaires were designed/deployed [9].

	1	2	3	4	5	
subitem not at all important	(•)	( )	( )	( )	( )	essential

Effacer la sélection

Does your paper address subitem 6a-i?

Copy and paste relevant sections from manuscript text

No online questionnaires were used.

6a-ii) Describe whether and how “use” (including intensity of use/dosage) was defined/measured/monitored

Describe whether and how “use” (including intensity of use/dosage) was defined/measured/monitored (logins, logfile analysis, etc.). Use/adoption metrics are important process outcomes that should be reported in any ehealth trial.

	1	2	3	4	5	
subitem not at all important	(●)	( )	( )	( )	( )	essential

Effacer la sélection

Does your paper address subitem 6a-ii?

Copy and paste relevant sections from manuscript text

Not applicable

6a-iii) Describe whether, how, and when qualitative feedback from participants was obtained

Describe whether, how, and when qualitative feedback from participants was obtained (e.g., through emails, feedback forms, interviews, focus groups).

	1	2	3	4	5	
subitem not at all important	( )	(●)	( )	( )	( )	essential

Effacer la sélection

Does your paper address subitem 6a-iii?

Copy and paste relevant sections from manuscript text

Participant qualitative feedback was collected as part of a prior feasibility study.

6b) Any changes to trial outcomes after the trial commenced, with reasons

Does your paper address CONSORT subitem 6b? *

Copy and paste relevant sections from the manuscript (include quotes in quotation marks "like this" to indicate direct quotes from your manuscript), or elaborate on this item by providing additional information not in the ms, or briefly explain why the item is not applicable/relevant for your study

No changes to the trial outcomes were made after trial commencement.

7a) How sample size was determined

NPT: When applicable, details of whether and how the clustering by care provides or centers was addressed

7a-i) Describe whether and how expected attrition was taken into account when calculating the sample size

Describe whether and how expected attrition was taken into account when calculating the sample size.

	1	2	3	4	5	
subitem not at all important	○	○	○	○	◉	essential

Effacer la sélection

Does your paper address subitem 7a-i?

Copy and paste relevant sections from manuscript title (include quotes in quotation marks "like this" to indicate direct quotes from your manuscript), or elaborate on this item by providing additional information not in the ms, or briefly explain why the item is not applicable/relevant for your study

"Drawing on findings from comparable studies, pilot data on the effects of VRT on AVH, and the small-to-moderate effect sizes typically observed for CBT, the between-group statistical power for the PSYRATS-AH outcome was estimated at f=0.25 [15, 16, 19, 41]. Sample size calculations were performed using G*Power. With a two-sided α=0.05 and 80% power, a sample of 52 participants per arm was required to detect differences using pre- and post-treatment assessments. To account for an anticipated 20% attrition rate (based on our pilot trial) and five degrees of freedom for potential confounders, the final recruitment target was set at 136 participants [16]."

7b) When applicable, explanation of any interim analyses and stopping guidelines

Does your paper address CONSORT subitem 7b? *

Copy and paste relevant sections from the manuscript (include quotes in quotation marks "like this" to indicate direct quotes from your manuscript), or elaborate on this item by providing additional information not in the ms, or briefly explain why the item is not applicable/relevant for your study

"Surveillance, Protocol Deviations, and Treatment Fidelity
Protocol deviations and undesirable treatment effects were compiled, analyzed, and presented by a graduate student (MB). Five categories of deviation were documented, including assessment timing, evaluator blinding, changes in antipsychotic medications, and treatment fidelity. The study was then overseen regularly by a steering committee that included the involved researchers, clinicians, a graduate student (MB), the president of the ethics committee, a biostatistician (CEG), and one patient-partner. After each of these meetings, the committee had to vote to continue the study, and the vote had to be unanimous to allow continuation. Moreover, the committee's mandate was to recommend future adjustments to the study, notably to minimize and manage protocol deviations. If serious adverse events had occurred, a protocol was in place to ensure they would have been handled seriously, including careful revision of the event by an independent committee to determine whether it was linked to therapy and what could have been done to prevent it, and the writing of a notice to the study Ethics committee."

8a) Method used to generate the random allocation sequence
NPT: When applicable, how care providers were allocated to each trial group

Does your paper address CONSORT subitem 8a? *

Copy and paste relevant sections from the manuscript (include quotes in quotation marks "like this" to indicate direct quotes from your manuscript), or elaborate on this item by providing additional information not in the ms, or briefly explain why the item is not applicable/relevant for your study

"After completion of the baseline assessment, participants were randomly assigned in a 1:1 ratio to VRT or CBT via an independent, secure, Internet-based service sequence generation process (https://www.sealedenvelope.com), using randomly varying block sizes of two, four, and six, stratified by gender and baseline clozapine treatment."

8b) Type of randomisation; details of any restriction (such as blocking and block size)

Does your paper address CONSORT subitem 8b? *

Copy and paste relevant sections from the manuscript (include quotes in quotation marks "like this" to indicate direct quotes from your manuscript), or elaborate on this item by providing additional information not in the ms, or briefly explain why the item is not applicable/relevant for your study

"After completion of the baseline assessment, participants were randomly assigned in a 1:1 ratio to VRT or CBT via an independent, secure, Internet-based service sequence generation process (https://www.sealedenvelope.com), using randomly varying block sizes of two, four, and six, stratified by gender and baseline clozapine treatment."

9) Mechanism used to implement the random allocation sequence (such as sequentially numbered containers), describing any steps taken to conceal the sequence until interventions were assigned

Does your paper address CONSORT subitem 9? *

Copy and paste relevant sections from the manuscript (include quotes in quotation marks "like this" to indicate direct quotes from your manuscript), or elaborate on this item by providing additional information not in the ms, or briefly explain why the item is not applicable/relevant for your study

"After completion of the baseline assessment, participants were randomly assigned in a 1:1 ratio to VRT or CBT via an independent, secure, Internet-based service sequence generation process (https://www.sealedenvelope.com), using randomly varying block sizes of two, four, and six, stratified by gender and baseline clozapine treatment."

10) Who generated the random allocation sequence, who enrolled participants, and who assigned participants to interventions

Does your paper address CONSORT subitem 10? *

Copy and paste relevant sections from the manuscript (include quotes in quotation marks "like this" to indicate direct quotes from your manuscript), or elaborate on this item by providing additional information not in the ms, or briefly explain why the item is not applicable/relevant for your study

"An independent statistician generated the random allocation sequence and was the only person with access to it and the block size. Once a participant was deemed eligible, the study coordinator (KP) entered the participant's gender and baseline clozapine treatment status into an internet-based randomization service, which generated the treatment allocation. The study coordinator then assigned the participant to the allocated intervention."

11a) If done, who was blinded after assignment to interventions (for example, participants, care providers, those assessing outcomes) and how

NPT: Whether or not administering co-interventions were blinded to group assignment

11a-i) Specify who was blinded, and who wasn’t

Specify who was blinded, and who wasn’t. Usually, in web-based trials it is not possible to blind the participants [1, 3] (this should be clearly acknowledged), but it may be possible to blind outcome assessors, those doing data analysis or those administering co-interventions (if any).

	1	2	3	4	5	
subitem not at all important	○	○	○	○	◉	essential

Effacer la sélection

Does your paper address subitem 11a-i? *

Copy and paste relevant sections from the manuscript (include quotes in quotation marks "like this" to indicate direct quotes from your manuscript), or elaborate on this item by providing additional information not in the ms, or briefly explain why the item is not applicable/relevant for your study

"Because the treatment was psychotherapeutic, neither patients nor therapists could be blinded. Nevertheless, outcome assessments were conducted by masked research assistants (unaware of group allocation) who did not have access to the collected data. To prevent unmasking, assessments and therapy were conducted in different buildings, and participants were reminded not to disclose their treatment allocation at the beginning of each assessment. In the rare event of accidental unmasking, which the evaluators flagged as soon as it occurred, questionnaires and scales requiring clinical judgment were rated from audio recordings by an independent evaluator blinded to treatment allocation. To avoid inter-rater variation, this recoding was performed across all assessment timepoints for the relevant participants."

11a-ii) Discuss e.g., whether participants knew which intervention was the "intervention of interest" and which one was the "comparator"

Informed consent procedures (4a-ii) can create biases and certain expectations - discuss e.g., whether participants knew which intervention was the "intervention of interest" and which one was the "comparator".

	1	2	3	4	5	
subitem not at all important	○	○	◉	○	○	essential

Effacer la sélection

Does your paper address subitem 11a-ii?

Copy and paste relevant sections from the manuscript (include quotes in quotation marks "like this" to indicate direct quotes from your manuscript), or elaborate on this item by providing additional information not in the ms, or briefly explain why the item is not applicable/relevant for your study

"Because the treatment was psychotherapeutic, neither patients nor therapists could be blinded."

11b) If relevant, description of the similarity of interventions

(this item is usually not relevant for ehealth trials as it refers to similarity of a placebo or sham intervention to a active medication/intervention)

Does your paper address CONSORT subitem 11b? *

Copy and paste relevant sections from the manuscript (include quotes in quotation marks "like this" to indicate direct quotes from your manuscript), or elaborate on this item by providing additional information not in the ms, or briefly explain why the item is not applicable/relevant for your study

Not applicable. The interventions were distinct psychotherapeutic interventions.

12a) Statistical methods used to compare groups for primary and secondary outcomes

NPT: When applicable, details of whether and how the clustering by care providers or centers was addressed

Does your paper address CONSORT subitem 12a? *

Copy and paste relevant sections from the manuscript (include quotes in quotation marks "like this" to indicate direct quotes from your manuscript), or elaborate on this item by providing additional information not in the ms, or briefly explain why the item is not applicable/relevant for your study

"Treatment efficacy and comparisons between treatments for primary and secondary outcomes were assessed using linear mixed models with maximum-likelihood estimation for missing data. Both between-group (treatment) and within-group (time) comparisons were examined, and time-by-treatment group interactions were used to see whether there was a significant change between VRT and CBT over time. Linear mixed models were chosen because they are well-established and recommended for analyzing psychotherapy RCTs with repeated measures. They handle unequal variances and correlated data, which are very common in treatment trials, and allow unequal numbers of follow-ups, making them suitable for clinical trial data. Furthermore, linear mixed models are based on maximum likelihood methods rather than the usual analysis of variance (ANOVA) methods. which require sphericity of the data and are not robust with small to moderate sample sizes [39, 40]."

12a-i) Imputation techniques to deal with attrition / missing values

Imputation techniques to deal with attrition / missing values: Not all participants will use the intervention/comparator as intended and attrition is typically high in ehealth trials. Specify how participants who did not use the application or dropped out from the trial were treated in the statistical analysis (a complete case analysis is strongly discouraged, and simple imputation techniques such as LOCF may also be problematic [4]).

	1	2	3	4	5	
subitem not at all important	○	○	○	○	◉	essential

Effacer la sélection

Does your paper address subitem 12a-i? *

Copy and paste relevant sections from the manuscript (include quotes in quotation marks "like this" to indicate direct quotes from your manuscript), or elaborate on this item by providing additional information not in the ms, or briefly explain why the item is not applicable/relevant for your study

"Treatment efficacy and comparisons between treatments for primary and secondary outcomes were assessed using linear mixed models with maximum-likelihood estimation for missing data. Both between-group (treatment) and within-group (time) comparisons were examined, and time-by-treatment group interactions were used to see whether there was a significant change between VRT and CBT over time. Linear mixed models were chosen because they are well-established and recommended for analyzing psychotherapy RCTs with repeated measures. They handle unequal variances and correlated data, which are very common in treatment trials, and allow unequal numbers of follow-ups, making them suitable for clinical trial data. Furthermore, linear mixed models are based on maximum likelihood methods rather than the usual analysis of variance (ANOVA) methods. which require sphericity of the data and are not robust with small to moderate sample sizes [39, 40]."

12b) Methods for additional analyses, such as subgroup analyses and adjusted analyses

Does your paper address CONSORT subitem 12b? *

Copy and paste relevant sections from the manuscript (include quotes in quotation marks "like this" to indicate direct quotes from your manuscript), or elaborate on this item by providing additional information not in the ms, or briefly explain why the item is not applicable/relevant for your study

To evaluate whether there were significant differences in antipsychotic use between the two groups at baseline, post-therapy, and 3-month follow-up, doses were converted to olanzapine equivalents and analyzed using the Mann–Whitney U test. The number of participants with a medication change in each group was compared using the chi-square test. Moreover, intervals between assessments and therapy sessions were also compared between the two groups using the Mann-Whitney U test.

X26) REB/IRB Approval and Ethical Considerations [recommended as subheading under "Methods"] (not a CONSORT item)

X26-i) Comment on ethics committee approval

	1	2	3	4	5	
subitem not at all important	○	○	○	○	◉	essential

Effacer la sélection

Does your paper address subitem X26-i?

Copy and paste relevant sections from the manuscript (include quotes in quotation marks "like this" to indicate direct quotes from your manuscript), or elaborate on this item by providing additional information not in the ms, or briefly explain why the item is not applicable/relevant for your study

"Ethical Considerations
The trial was conducted in accordance with the Declaration of Helsinki. It was approved by the institutional ethical committee of the Centre intégré universitaire de santé et de services sociaux (CIUSSS) de l'Est-de-l'Île-de-Montréal (project number: MP-12-2019-1726) in April 2019."

x26-ii) Outline informed consent procedures

Outline informed consent procedures e.g., if consent was obtained offline or online (how? Checkbox, etc.?), and what information was provided (see 4a-ii). See [6] for some items to be included in informed consent documents.

	1	2	3	4	5	
subitem not at all important	○	○	○	○	◉	essential

Effacer la sélection

Does your paper address subitem X26-ii?

Copy and paste relevant sections from the manuscript (include quotes in quotation marks "like this" to indicate direct quotes from your manuscript), or elaborate on this item by providing additional information not in the ms, or briefly explain why the item is not applicable/relevant for your study

"The trial was conducted in accordance with the Declaration of Helsinki. It was approved by the institutional ethical committee of the Centre intégré universitaire de santé et de services sociaux (CIUSSS) de l'Est-de-l'Île-de-Montréal (project number: MP-12-2019-1726) in April 2019. Prior to their inclusion in the study, the Information and Consent Form was carefully presented and explained to potential participants by a research assistant or psychiatric nurse. The paper form was also provided, along with time and space to make an informed decision. All questions were answered as needed, whether during the presentation or later by phone. Capacity to consent was also assessed; for example, participants were asked to summarise different sections of the form. If they agreed to participate and had capacity, they were allowed to provide written informed consent, and basic information was collected during the same visit, including sociodemographic data, contact information, and whether they were currently using clozapine (for randomization purposes)."

X26-iii) Safety and security procedures

Safety and security procedures, incl. privacy considerations, and any steps taken to reduce the likelihood or detection of harm (e.g., education and training, availability of a hotline)

	1	2	3	4	5	
subitem not at all important	○	○	○	○	●	essential

Effacer la sélection

Does your paper address subitem X26-iii?

Copy and paste relevant sections from the manuscript (include quotes in quotation marks "like this" to indicate direct quotes from your manuscript), or elaborate on this item by providing additional information not in the ms, or briefly explain why the item is not applicable/relevant for your study

"Participants were provided access to a 24/7 helpline staffed by an unblinded psychiatric nurse, enabling them to discuss any concerns, adverse effects, or difficulties arising between sessions as needed."
"The study was then overseen regularly by a steering committee that included the involved researchers, clinicians, a graduate student (MB), the president of the ethics committee, a biostatistician (CEG), and one patient-partner. After each of these meetings, the committee had to vote to continue the study, and the vote had to be unanimous to allow continuation. Moreover, the committee's mandate was to recommend future adjustments to the study, notably to minimize and manage protocol deviations. If serious adverse events had occurred, a protocol was in place to ensure they would have been handled seriously, including careful revision of the event by an independent committee to determine whether it was linked to therapy and what could have been done to prevent it, and the writing of a notice to the study Ethics committee."

RESULTS

13a) For each group, the numbers of participants who were randomly assigned, received intended treatment, and were analysed for the primary outcome

NPT: The number of care providers or centers performing the intervention in each group and the number of patients treated by each care provider in each center

Does your paper address CONSORT subitem 13a? *

Copy and paste relevant sections from the manuscript (include quotes in quotation marks "like this" to indicate direct quotes from your manuscript), or elaborate on this item by providing additional information not in the ms, or briefly explain why the item is not applicable/relevant for your study

"Following randomization, 67 participants were allocated to the VRT condition and 69 to the short course CBT condition."

13b) For each group, losses and exclusions after randomisation, together with reasons

Does your paper address CONSORT subitem 13b? (NOTE: Preferably, this is shown in a CONSORT flow diagram) *

Copy and paste relevant sections from the manuscript (include quotes in quotation marks "like this" to indicate direct quotes from your manuscript), or elaborate on this item by providing additional information not in the ms, or briefly explain why the item is not applicable/relevant for your study

"Figure 1. CONSORT diagram of all participants who were assessed for eligibility, randomized to Virtual Reality-based Therapy or Cognitive Behavioral Therapy, and completed therapy, post-therapy assessment, and 3-month follow-up assessment."

13b-i) Attrition diagram

Strongly recommended: An attrition diagram (e.g., proportion of participants still logging in or using the intervention/comparator in each group plotted over time, similar to a survival curve) or other figures or tables demonstrating usage/dose/engagement.

	1	2	3	4	5	
subitem not at all important	○	○	○	○	◉	essential

Effacer la sélection

Does your paper address subitem 13b-i?

Copy and paste relevant sections from the manuscript or cite the figure number if applicable (include quotes in quotation marks "like this" to indicate direct quotes from your manuscript), or elaborate on this item by providing additional information not in the ms, or briefly explain why the item is not applicable/relevant for your study

"Figure 1. CONSORT diagram of all participants who were assessed for eligibility, randomized to Virtual Reality-based Therapy or Cognitive Behavioral Therapy, and completed therapy, post-therapy assessment, and 3-month follow-up assessment."

14a) Dates defining the periods of recruitment and follow-up

Does your paper address CONSORT subitem 14a? *

Copy and paste relevant sections from the manuscript (include quotes in quotation marks "like this" to indicate direct quotes from your manuscript), or elaborate on this item by providing additional information not in the ms, or briefly explain why the item is not applicable/relevant for your study

"Participant recruitment occurred between April 2019 and June 2025."
"Therapy sessions were delivered between April 2019 and October 2025, and the final 3-month follow-up assessment was completed in January 2026."

14a-i) Indicate if critical “secular events” fell into the study period

Indicate if critical “secular events” fell into the study period, e.g., significant changes in Internet resources available or “changes in computer hardware or Internet delivery resources”

	1	2	3	4	5	
subitem not at all important	○	○	○	●	○	essential

Effacer la sélection

Does your paper address subitem 14a-i?

Copy and paste relevant sections from the manuscript (include quotes in quotation marks "like this" to indicate direct quotes from your manuscript), or elaborate on this item by providing additional information not in the ms, or briefly explain why the item is not applicable/relevant for your study

No secular events or changes to the hardware, software, internet resources, or intervention delivery platform occurred during the study period.

14b) Why the trial ended or was stopped (early)

Does your paper address CONSORT subitem 14b? *

Copy and paste relevant sections from the manuscript (include quotes in quotation marks "like this" to indicate direct quotes from your manuscript), or elaborate on this item by providing additional information not in the ms, or briefly explain why the item is not applicable/relevant for your study

The trial was completed as planned and was not stopped early.

15) A table showing baseline demographic and clinical characteristics for each group

NPT: When applicable, a description of care providers (case volume, qualification, expertise, etc.) and centers (volume) in each group

Does your paper address CONSORT subitem 15? *

Copy and paste relevant sections from the manuscript (include quotes in quotation marks "like this" to indicate direct quotes from your manuscript), or elaborate on this item by providing additional information not in the ms, or briefly explain why the item is not applicable/relevant for your study

Table 1. Baseline sociodemographic and clinical characteristics.

15-i) Report demographics associated with digital divide issues

In ehealth trials it is particularly important to report demographics associated with digital divide issues, such as age, education, gender, social-economic status, computer/Internet/ehealth literacy of the participants, if known.

	1	2	3	4	5	
subitem not at all important	(●)	○	○	○	○	essential

Effacer la sélection

Does your paper address subitem 15-i? *

Copy and paste relevant sections from the manuscript (include quotes in quotation marks "like this" to indicate direct quotes from your manuscript), or elaborate on this item by providing additional information not in the ms, or briefly explain why the item is not applicable/relevant for your study

Digital divide–related characteristics were not assessed because the interventions were therapist-delivered.

16) For each group, number of participants (denominator) included in each analysis and whether the analysis was by original assigned groups

16-i) Report multiple “denominators” and provide definitions

Report multiple “denominators” and provide definitions: Report N’s (and effect sizes) “across a range of study participation [and use] thresholds” [1], e.g., N exposed, N consented, N used more than x times, N used more than y weeks, N participants “used” the intervention/comparator at specific pre-defined time points of interest (in absolute and relative numbers per group). Always clearly define “use” of the intervention.

	1	2	3	4	5	
subitem not at all important	○	○	○	◉	○	essential

Effacer la sélection

Does your paper address subitem 16-i? *

Copy and paste relevant sections from the manuscript (include quotes in quotation marks "like this" to indicate direct quotes from your manuscript), or elaborate on this item by providing additional information not in the ms, or briefly explain why the item is not applicable/relevant for your study

"Among completers, 51 participants completed nine 1-hour VRT sessions, each including 5 to 20 minutes of VR immersion, for a total of 459 therapy sessions. In comparison, 53 participants completed nine 1-hour CBT sessions, for a total of 477 therapy sessions."

16-ii) Primary analysis should be intent-to-treat

Primary analysis should be intent-to-treat, secondary analyses could include comparing only "users", with the appropriate caveats that this is no longer a randomized sample (see 18-i).

	1	2	3	4	5	
subitem not at all important	○	○	○	○	◉	essential

Effacer la sélection

Does your paper address subitem 16-ii?

Copy and paste relevant sections from the manuscript (include quotes in quotation marks "like this" to indicate direct quotes from your manuscript), or elaborate on this item by providing additional information not in the ms, or briefly explain why the item is not applicable/relevant for your study

"For the primary outcome of auditory hallucination severity, the intention-to-treat analysis using linear mixed models revealed a significant time-by-treatment interaction measured by PSYRATS-AH total score (p=0.012)."

17a) For each primary and secondary outcome, results for each group, and the estimated effect size and its precision (such as 95% confidence interval)

Does your paper address CONSORT subitem 17a? *

Copy and paste relevant sections from the manuscript (include quotes in quotation marks "like this" to indicate direct quotes from your manuscript), or elaborate on this item by providing additional information not in the ms, or briefly explain why the item is not applicable/relevant for your study

"Table 2. Longitudinal effects of Virtual Reality-based Therapy vs Cognitive Behavioral Therapy on auditory hallucination severity and related clinical outcomes using linear mixed models. n = 136"

17a-i) Presentation of process outcomes such as metrics of use and intensity of use

In addition to primary/secondary (clinical) outcomes, the presentation of process outcomes such as metrics of use and intensity of use (dose, exposure) and their operational definitions is critical. This does not only refer to metrics of attrition (13-b) (often a binary variable), but also to more continuous exposure metrics such as “average session length”. These must be accompanied by a technical description how a metric like a “session” is defined (e.g., timeout after idle time) [1] (report under item 6a).

	1	2	3	4	5	
subitem not at all important	○	○	○	◉	○	essential

Effacer la sélection

Does your paper address subitem 17a-i?

Copy and paste relevant sections from the manuscript (include quotes in quotation marks "like this" to indicate direct quotes from your manuscript), or elaborate on this item by providing additional information not in the ms, or briefly explain why the item is not applicable/relevant for your study

"Among completers, 51 participants completed nine 1-hour VRT sessions, each including 5 to 20 minutes of VR immersion, for a total of 459 therapy sessions. In comparison, 53 participants completed nine 1-hour CBT sessions, for a total of 477 therapy sessions."

17b) For binary outcomes, presentation of both absolute and relative effect sizes is recommended

Does your paper address CONSORT subitem 17b? *

Copy and paste relevant sections from the manuscript (include quotes in quotation marks "like this" to indicate direct quotes from your manuscript), or elaborate on this item by providing additional information not in the ms, or briefly explain why the item is not applicable/relevant for your study

There were no binary outcomes in this study.

18) Results of any other analyses performed, including subgroup analyses and adjusted analyses, distinguishing pre-specified from exploratory

Does your paper address CONSORT subitem 18? *

Copy and paste relevant sections from the manuscript (include quotes in quotation marks "like this" to indicate direct quotes from your manuscript), or elaborate on this item by providing additional information not in the ms, or briefly explain why the item is not applicable/relevant for your study

"Protocol deviations were prospectively recorded and reviewed during steering committee meetings.
First, deviations in assessment timing were generally low. The interval between the initial session and the baseline assessment exceeded the 7-day threshold for a greater proportion of participants in the VRT group; similarly, a greater number of individuals in the CBT group required more time to complete the 3-month follow-up assessment. The other intervals were comparable, without statistically significant differences. Full details were provided in Table S2 (Multimedia Appendix).
Second, evaluator blinding was compromised in three post-treatment assessments, in which case outcomes requiring clinical judgment were re-rated from audio recordings by an independent blinded evaluator for those participants.
Third, changes in antipsychotic medication were common, occurring in 24.8% of participants during therapy and in 50% of participants by the 3-month follow-up visit. However, no between-group differences were observed (please refer to Table S3 in Multimedia Appendix for details). In addition, the olanzapine-equivalent dosage in antipsychotic medication did not differ between the two treatment arms at any of the three assessments, as detailed in Table S4 (Multimedia Appendix)."

18-i) Subgroup analysis of comparing only users

A subgroup analysis of comparing only users is not uncommon in ehealth trials, but if done, it must be stressed that this is a self-selected sample and no longer an unbiased sample from a randomized trial (see 16-iii).

	1	2	3	4	5	
subitem not at all important	○	◉	○	○	○	essential

Effacer la sélection

Does your paper address subitem 18-i?

Copy and paste relevant sections from the manuscript (include quotes in quotation marks "like this" to indicate direct quotes from your manuscript), or elaborate on this item by providing additional information not in the ms, or briefly explain why the item is not applicable/relevant for your study

No completer-only subgroup analysis was performed.

19) All important harms or unintended effects in each group
(for specific guidance see CONSORT for harms)

Does your paper address CONSORT subitem 19? *

Copy and paste relevant sections from the manuscript (include quotes in quotation marks "like this" to indicate direct quotes from your manuscript), or elaborate on this item by providing additional information not in the ms, or briefly explain why the item is not applicable/relevant for your study

"Undesirable effects
Negative effects were reported by 100 out of 136 participants. Missing data mainly occurred when participants were unreachable, which was also common among excluded participants and those lost to follow-up. Although efforts were made to contact excluded participants once their mental state stabilized, the questionnaire was not conducted if they were unable to properly recall therapy. Reported negative effects were low and comparable across study conditions. Results by subscale were presented in Table 3. Additional information is presented in Figure S2 (Multimedia Appendix).
[Please insert Table 3 about here.]
Other potentially serious adverse events were thoroughly examined. Hospital admissions were infrequent, but more prevalent in the CBT group (7 admissions for psychological health events and 1 for physical health in the CBT group, versus only 1 psychiatric admission in the VRT group). One detox admission occurred in the CBT group. Finally, one death occurred in the CBT group due to medical causes unrelated to the trial protocol."

19-i) Include privacy breaches, technical problems

Include privacy breaches, technical problems. This does not only include physical "harm" to participants, but also incidents such as perceived or real privacy breaches [1], technical problems, and other unexpected/unintended incidents. "Unintended effects" also includes unintended positive effects [2].

	1	2	3	4	5	
subitem not at all important	○	○	◉	○	○	essential

Effacer la sélection

Does your paper address subitem 19-i?

Copy and paste relevant sections from the manuscript (include quotes in quotation marks "like this" to indicate direct quotes from your manuscript), or elaborate on this item by providing additional information not in the ms, or briefly explain why the item is not applicable/relevant for your study

No privacy breaches or technical problems occurred during the trial.

19-ii) Include qualitative feedback from participants or observations from staff/researchers

Include qualitative feedback from participants or observations from staff/researchers, if available, on strengths and shortcomings of the application, especially if they point to unintended/unexpected effects or uses. This includes (if available) reasons for why people did or did not use the application as intended by the developers.

	1	2	3	4	5	
subitem not at all important	○	○	◉	○	○	essential

Effacer la sélection

Does your paper address subitem 19-ii?

Copy and paste relevant sections from the manuscript (include quotes in quotation marks "like this" to indicate direct quotes from your manuscript), or elaborate on this item by providing additional information not in the ms, or briefly explain why the item is not applicable/relevant for your study

Qualitative feedback from participants was collected in a previous feasibility study.

DISCUSSION

22) Interpretation consistent with results, balancing benefits and harms, and considering other relevant evidence

NPT: In addition, take into account the choice of the comparator, lack of or partial blinding, and unequal expertise of care providers or centers in each group

22-i) Restate study questions and summarize the answers suggested by the data, starting with primary outcomes and process outcomes (use)

Restate study questions and summarize the answers suggested by the data, starting with primary outcomes and process outcomes (use).

	1	2	3	4	5	
subitem not at all important	○	○	○	○	◉	essential

Effacer la sélection

Does your paper address subitem 22-i? *

Copy and paste relevant sections from the manuscript (include quotes in quotation marks "like this" to indicate direct quotes from your manuscript), or elaborate on this item by providing additional information not in the ms, or briefly explain why the item is not applicable/relevant for your study

"This assessor-blind RCT aimed to determine whether VRT was superior to a short course CBT in reducing AVH. To our knowledge, this is the first fully powered RCT to compare an evidence-based therapy (CBT) with VRT in patients with treatment-resistant schizophrenia. Relative to CBT, VRT produced significantly greater and more enduring reductions in overall AVH severity, with benefits maintained and further consolidated at the 3-month follow-up visit. These findings support the superiority of VRT over CBT for reducing AVH severity, thereby confirming the primary hypothesis. Notably, VRT exhibited a large effect size at post-treatment on the primary outcome (d=0.81), which increased at the 3-month follow-up (d=1.17), indicating not only a robust short-term impact but also a progressive consolidation of therapeutic gains over time. In contrast, CBT yielded a moderately significant reduction at post-treatment (d=0.58), and no significant improvement at 3-month follow-up (d=0.39), suggesting an attenuation of early gains. Consistent with these differential trajectories, VRT significantly outperformed CBT on the primary outcome of reducing AVH severity across time ($p<0.05$). This pattern aligns with findings typically reported in CBT trials, in which reductions in hallucinations tend to be modest and often wane at follow-up [10].
Secondary outcomes highlighted meaningful psychological benefits of VRT, consistent with most previous trials [16, 18]. Significant within-group impacts were seen on all auditory hallucination subscales, including voice-associated distress, frequency, and attribution. These findings suggest that VRT not only helped participants manage their emotions but also had an impact on how frequent and disturbing the hallucinations could be. Although it was not the main target of therapy, reductions in delusion-associated distress and frequency were also observed. More importantly, VRT results over time were significantly superior to those of CBT for delusion-associated distress, possibly because these delusions were frequently linked to the content of the voices. Significant effects were observed on voice-related beliefs, specifically omnipotence, as well as on voice acceptance, indicating broader shifts in the relational schema toward voices. Furthermore, VRT was associated with significant reductions in psychotic symptoms, as well as improvements in self-esteem and emotion regulation. The changes observed in psychotic symptoms (Positive And Negative Syndrome Scale total and positive subscale scores) are consistent with the existing psychotherapeutic literature on psychosis (e.g., Turner et al., 2025 [45])."

22-ii) Highlight unanswered new questions, suggest future research

Highlight unanswered new questions, suggest future research.

	1	2	3	4	5	
subitem not at all important	○	○	○	○	◉	essential

Effacer la sélection

Does your paper address subitem 22-ii?

Copy and paste relevant sections from the manuscript (include quotes in quotation marks "like this" to indicate direct quotes from your manuscript), or elaborate on this item by providing additional information not in the ms, or briefly explain why the item is not applicable/relevant for your study

"Future studies should incorporate extended follow-up periods to assess whether the benefits of VRT are sustained over time. Moreover, similar interventions or techniques using VR could also be developed or integrated into this version of VRT in order to tackle the other components of psychosis, such as delusions, social skills, or negative symptoms. Further research into the mechanisms of action underlying VRT is needed to identify its active therapeutic components and inform ongoing intervention refinement. Health-economic analyses will be essential for evaluating the cost-effectiveness of VRT and supporting its potential adoption within publicly funded mental health services. Finally, implementation projects are warranted to assess the feasibility and scalability of VRT across diverse real-world clinical settings, thereby facilitating its adoption in routine care pathways."

20) Trial limitations, addressing sources of potential bias, imprecision, and, if relevant, multiplicity of analyses

20-i) Typical limitations in ehealth trials

Typical limitations in ehealth trials: Participants in ehealth trials are rarely blinded. Ehealth trials often look at a multiplicity of outcomes, increasing risk for a Type I error. Discuss biases due to non-use of the intervention/usability issues, biases through informed consent procedures, unexpected events.

	1	2	3	4	5	
subitem not at all important	○	○	○	○	◉	essential

Effacer la sélection

Does your paper address subitem 20-i? *

Copy and paste relevant sections from the manuscript (include quotes in quotation marks "like this" to indicate direct quotes from your manuscript), or elaborate on this item by providing additional information not in the ms, or briefly explain why the item is not applicable/relevant for your study

"Despite numerous strengths, a few limitations must be acknowledged. Due to the COVID-19 pandemic, CBT was delivered remotely to 35.8% of patients. In contrast, VRT remained in-person, as this modality required digital materials and could not be delivered remotely. This discrepancy in delivery format may have affected the comparability of the interventions. However, the primary outcome scores (PSYRATS-AH) did not differ significantly between those who received in-person vs remote CBT at post-therapy (p=0.709) and at the 3-month follow-up (p=0.919). In addition, meta-analyses have demonstrated that CBT is equally effective when delivered in-person or remotely, suggesting that this modality change is unlikely to have substantially affected the study outcomes [25]. In addition, the COVID-19 pandemic did not increase the number of participants with protocol deviations, but during this period, the longest delays between assessments were observed in both study arms. To minimize these delays, some evaluations were conducted remotely via videoconference. Although patients and their treating teams were consulted to identify planned medication changes up to the post-therapy assessment for patient eligibility, unplanned medication changes still occurred in a subset of patients (22.9% for VRT and 26.4% for CBT). However, the proportion of patients who experienced medication changes did not differ significantly between the two groups. Additionally, these changes reflect typical real-world clinical practice and may enhance the generalizability of the findings."

21) Generalisability (external validity, applicability) of the trial findings

NPT: External validity of the trial findings according to the intervention, comparators, patients, and care providers or centers involved in the trial

21-i) Generalizability to other populations

Generalizability to other populations: In particular, discuss generalizability to a general Internet population, outside of a RCT setting, and general patient population, including applicability of the study results for other organizations

	1	2	3	4	5	
subitem not at all important	○	○	○	○	◉	essential

Effacer la sélection

Does your paper address subitem 21-i?

Copy and paste relevant sections from the manuscript (include quotes in quotation marks "like this" to indicate direct quotes from your manuscript), or elaborate on this item by providing additional information not in the ms, or briefly explain why the item is not applicable/relevant for your study

"Finally, the generalizability of these findings should be considered in light of the study population, which was restricted to individuals with schizophrenia or schizoaffective disorder experiencing treatment-resistant AVH. Nevertheless, previous studies conducted by other research groups have reported positive outcomes after extending Avatar Therapy to broader populations with psychotic disorders, suggesting that the intervention may be applicable beyond the specific population included in the present study [18, 21]. In addition, although therapy sessions were conducted in Montreal, participants were recruited from inpatient and outpatient services and came from both urban and suburban areas, increasing the diversity of the study sample."

21-ii) Discuss if there were elements in the RCT that would be different in a routine application setting

Discuss if there were elements in the RCT that would be different in a routine application setting (e.g., prompts/reminders, more human involvement, training sessions or other co-interventions) and what impact the omission of these elements could have on use, adoption, or outcomes if the intervention is applied outside of a RCT setting.

	1	2	3	4	5	
subitem not at all important	○	○	○	○	◉	essential

Effacer la sélection

Does your paper address subitem 21-ii?

Copy and paste relevant sections from the manuscript (include quotes in quotation marks "like this" to indicate direct quotes from your manuscript), or elaborate on this item by providing additional information not in the ms, or briefly explain why the item is not applicable/relevant for your study

"However, the proportion of patients who experienced medication changes did not differ significantly between the two groups. Additionally, these changes reflect typical real-world clinical practice and may enhance the generalizability of the findings. Another limitation concerns the controlled nature of the trial, as eligibility criteria and the number of therapy sessions may be adapted to individual clinical needs in routine practice."

OTHER INFORMATION

23) Registration number and name of trial registry

Does your paper address CONSORT subitem 23? *

Copy and paste relevant sections from the manuscript (include quotes in quotation marks "like this" to indicate direct quotes from your manuscript), or elaborate on this item by providing additional information not in the ms, or briefly explain why the item is not applicable/relevant for your study

"Registration
ClinicalTrials.gov [NCT04054778]; https://clinicaltrials.gov/."

24) Where the full trial protocol can be accessed, if available

Does your paper address CONSORT subitem 24? *

Cite a Multimedia Appendix, other reference, or copy and paste relevant sections from the manuscript (include quotes in quotation marks "like this" to indicate direct quotes from your manuscript), or elaborate on this item by providing additional information not in the ms, or briefly explain why the item is not applicable/relevant for your study

https://cdn.clinicaltrials.gov/large-docs/78/NCT04054778/Prot_SAP_000.pdf

25) Sources of funding and other support (such as supply of drugs), role of funders

Does your paper address CONSORT subitem 25? *

Copy and paste relevant sections from the manuscript (include quotes in quotation marks "like this" to indicate direct quotes from your manuscript), or elaborate on this item by providing additional information not in the ms, or briefly explain why the item is not applicable/relevant for your study

"This study was funded by the Canadian Institutes of Health Research (CIHR; grant no. 159540), the J.-Louis Lévesque Foundation, and the Institut universitaire en santé mentale de Montréal Foundation. Additional support was provided by the Direction of Mental Health, Addiction, and Homelessness Programs at the Institut universitaire en santé mentale de Montréal. With respect to individual funding: Dr. Beaudoin held doctoral scholarships from the CIHR and the Fonds de recherche du Québec–Santé (FRQS), Mrs. Giguère held a doctoral scholarship from the CIHR, Dr. Dumais holds a senior salary award from the FRQS, and Prof. Potvin holds the Eli Lilly Canada Chair on Schizophrenia Research. The funding bodies had no role in study design, data collection, data analysis, data interpretation, or manuscript preparation."

X27) Conflicts of Interest (not a CONSORT item)

X27-i) State the relation of the study team towards the system being evaluated

In addition to the usual declaration of interests (financial or otherwise), also state the relation of the study team towards the system being evaluated, i.e., state if the authors/evaluators are distinct from or identical with the developers/sponsors of the intervention.

	1	2	3	4	5	
subitem not at all important	○	○	○	○	●	essential

Effacer la sélection

Does your paper address subitem X27-i?

Copy and paste relevant sections from the manuscript (include quotes in quotation marks "like this" to indicate direct quotes from your manuscript), or elaborate on this item by providing additional information not in the ms, or briefly explain why the item is not applicable/relevant for your study

"The trial coordinator, Mrs. Phraxayavong received a salary from AD Psychiatric Services and Research, a private medical company owned by the principal investigator, Dr. Alexandre Dumais. Dr. Emmanuel Stip declares the following competing interests: lecturing fees from Otsuka; advisory board membership with Johnson & Johnson and AbbVie; and board membership with Les Impatients. It should be noted that Dr. Stip received no compensation for the present trial. The VR platform used in this study was developed by OVA and is owned by AD Psychiatric Services. All remaining authors declare no conflicts of interest."

About the CONSORT EHEALTH checklist

As a result of using this checklist, did you make changes in your manuscript? *

- ○ yes, major changes
- ◉ yes, minor changes
- ○ no

What were the most important changes you made as a result of using this checklist?

More detailed information is provided in the Abstract, Methods, and Results sections.

How much time did you spend on going through the checklist INCLUDING making changes in your manuscript *

6 hours

Your answer must have a minimum of 25 characters.

As a result of using this checklist, do you think your manuscript has improved? *

- (x) yes
- ( ) no
- ( ) Autre :

Would you like to become involved in the CONSORT EHEALTH group?

This would involve for example becoming involved in participating in a workshop and writing an "Explanation and Elaboration" document

- ( ) yes
- (x) no
- ( ) Autre :

Effacer la sélection

Any other comments or questions on CONSORT EHEALTH

Votre réponse

?

STOP - Save this form as PDF before you click submit

To generate a record that you filled in this form, we recommend to generate a PDF of this page (on a Mac, simply select "print" and then select "print as PDF") before you submit it.

When you submit your (revised) paper to JMIR, please upload the PDF as supplementary file.

Don't worry if some text in the textboxes is cut off, as we still have the complete information in our database. Thank you!

Final step: Click submit !

Click submit so we have your answers in our database!

Envoyer

Effacer le formulaire

N'envoyez jamais de mots de passe via Google Forms.

Ce contenu n'est ni rédigé, ni cautionné par Google. - Contacter le propriétaire du formulaire - Conditions d'utilisation - Règles de confidentialité

Ce formulaire vous semble suspect ? Signaler

Google Forms
