# Supplementary material for: Virtual Reality–Assisted Therapy Compared to Cognitive Behavioral Therapy for Patients With Treatment-Resistant Schizophrenia: Assessor-Blind Randomized Controlled Trial
Source: JMIR Ment Health. 2026 Sep 16;13:e106791. doi: 10.2196/106791 (PMC13580698; doi:10.2196/106791)
Supplement: Supplementary file 2 [file mental-v13-e106791-s001.docx]

**Multimedia Appendix**

**Virtual Reality-Assisted Therapy vs Cognitive Behavioral Therapy for Treatment-Resistant Schizophrenia: Randomized Controlled Trial**

Supplementary figures and tables.

**Figure S1.** Setting of the immersive sessions.

**Figure S2.** Number of patients reporting each item on the Negative Effects Questionnaire for Avatar therapy (n=50) and Cognitive behavioral therapy (n=50).

**Table S1.** Summary of the questionnaires used in the present study, including their psychometric properties, score range, and subscales.

**Table S2.** Between-group comparison of overall retention, dropouts, exclusions and missing data in participants randomized to complete either virtual reality-assisted therapy (VRT; n=67) or a short course of cognitive behavioral therapy (CBT; n=69).

**Table S3.** Within-group longitudinal changes following Virtual Reality-assisted Therapy and Cognitive Behavioral Therapy on auditory hallucination severity and related clinical outcomes using linear mixed models (n=136).

**Table S4.** Protocol deviations related to therapy duration and assessment intervals

**Table S5.** Protocol deviations related to antipsychotic treatment changes during the study

**Table S6.** Equivalent olanzapine dosage at baseline, post-therapy, and 3-month follow-up

**Table S7.** Evolution of feeling of presence over the course of Virtual Reality-assisted Therapy as measured using a graphic rating scale and the Igroup Presence Questionnaire.

**Figure S1.** Setting of the immersive sessions. The participant, wearing a virtual reality headset and noise-canceling headphones, sat in an adjacent room and was invited to enter a dialogue with their avatar in the VR environment. The therapist could observe the participant through a one-way mirror and monitor, on a computer screen, what the participant was seeing in the VR headset. Explicit consent was obtained from the individuals in this figure to use their image for publication.

Number of participants reporting the negative effect

|  | VRT | CBT |
| --- | --- | --- |
| Item 1: Sleep problems | 6 | 4 |
| Item 2: Increased stress | 14 | 8 |
| Item 3: Increased anxiety | 12 | 7 |
| Item 4: Heightened worry | 6 | 3 |
| Item 5: Feeling dejected | 2 | 3 |
| Item 6: Hopelessness | 4 | 5 |
| Item 7: Lower self‑esteem | 0 | 2 |
| Item 8: Loss of self‑confidence | 0 | 1 |
| Item 9: Increased sadness | 7 | 5 |
| Item 10: Reduced competence | 2 | 1 |
| Item 11: Unpleasant emotions | 10 | 7 |
| Item 12: Symptom worsening | 2 | 3 |
| Item 13: Resurfacing memories | 18 | 12 |
| Item 14: Fear of stigma | 1 | 4 |
| Item 15: Suicidal thoughts | 1 | 1 |
| Item 16: Shame about treatment | 1 | 3 |
| Item 17: Loss of optimism | 3 | 6 |
| Item 18: Belief improvement is impossible | 2 | 6 |
| Item 19: Loss of hope in help | 2 | 3 |
| Item 20: Dependency on treatment | 8 | 5 |
| Item 21: Dependency on therapist | 9 | 6 |
| Item 22: Not understanding treatment | 15 | 14 |
| Item 23: Not understanding therapist | 8 | 9 |
| Item 24: Low confidence in treatment | 7 | 7 |
| Item 25: Low confidence in therapist | 0 | 2 |
| Item 26: No perceived results | 3 | 14 |
| Item 27: Unmet treatment expectations | 4 | 15 |
| Item 28: Unmet therapist expectations | 0 | 4 |
| Item 29: Poor treatment quality | 1 | 2 |
| Item 30: Treatment not suitable | 2 | 12 |
| Item 31: Weak therapeutic bond | 2 | 5 |
| Item 32: Treatment not motivating | 2 | 12 |

**Figure S2.** Number of participants reporting each item and attributing it partially or entirely to the study treatment on the Negative Effects Questionnaire for Virtual Reality-assisted Therapy(n=50) and a short course of Cognitive Behavioral Therapy (n=50).

**Table S1.** Summary of the questionnaires used in the present study, including their psychometric properties, score range, and subscales.

| Clinical variable | Tool | Description | Validation in a psychotic or related population |
| --- | --- | --- | --- |
| Psychiatric diagnoses | Structured Clinical Interview for the Diagnostic (SCID) | The SCID is a semi-structured interview that provides diagnoses based on the Diagnostic and Statistical Manual of Mental Disorders, Fifth Edition, criteria and requires the interviewer’s clinical judgment. It was used to confirm the psychiatric diagnoses of schizophrenia and schizoaffective disorder. | The SCID for the Schizophrenia spectrum and other psychotic disorders demonstrates almost complete agreement with the gold-standard diagnosis (κ=0.90), excellent sensitivity (0.89) and specificity (0.98), as well as excellent inter-rater reliability [1]. |
| Sociodemographics | Sociodemographic questionnaire | Sociodemographic characteristics, including age, sex, marital status, education, employment, and ethnicity, were assessed using a sociodemographic questionnaire adapted from items drawn from the Prevention Status Reports Toolkit, the Canadian Community Health Survey, and the Canadian Census of Population. | N/A |
| Psychotic symptoms | Psychotic Symptoms Rating Scale (PSYRATS) | The PSYRATS is a 17-item, semi-structured, clinician-rated interview designed to assess the severity of auditory hallucinations and delusions over the past 7 days. Each item is scored from 0 to 4, yielding a total score ranging from 0 to 68. The scale is divided into two subscales: auditory hallucinations (11 items) and delusions (6 items). The Auditory Hallucinations subscale quantifies multiple phenomenological dimensions of auditory hallucinations, including frequency, loudness, voice-associated distress, and attribution (i.e., beliefs about the origin of the voices). The Delusions subscale includes items assessing the frequency of delusions and delusion-associated distress. Higher scores indicate greater severity of psychotic symptoms, whereas lower scores reflect lower symptom severity [2]. | The PSYRATS have shown excellent interrater reliability (ICC=0.9) and good validity [2]. |
| Beliefs About Voices | Beliefs About Voices Questionnaire-Revised (BAVQ-R) | The BAVQ-R is a 35-item self-report questionnaire that assesses patients’ beliefs, emotions, and behaviors regarding auditory hallucinations over the past 7 days. Each item is scored from 0 to 3, yielding a total score ranging from 0 to 105. The scale comprises three belief-related subscales: malevolence, benevolence, and omnipotence, and two subscales related to emotional and behavioral responses to auditory hallucinations: resistance and engagement [3]. | The Cronbach’s α for the total scale has been found to be high (mean α = 0.86. Factor analysis supports its five subscales, with Cronbach’s α ranging from 0.74 to 0.88 [3]. For construct validity, malevolence, omnipotence, and resistance were intercorrelated (r=0.56–0.64, p<0.001), while benevolence and engagement were also correlated (r=0.62, p<0.001) [3]. |
| Acceptance-based attitudes and behaviours toward voice | Voices Acceptance and Action Scale (VASS) | This self-report measure assessed acceptance of and disengagement from auditory and command hallucinations, as well as commitment to effective action rather than acting in response to the voice. The questionnaire uses a 31-item Likert scale, with each item scored from 0 to 4, yielding a total score ranging from 31 to 124 [4]. | The VASS has an excellent test-retest reliability (ICC=0.73 and good construct validity. The Cronbach’s α for the total scale has been found to be high (α =0.90) [4]. |
| Symptoms of schizophrenia | Positive And Negative Syndrome Scale (PANSS) | The PANSS is a 30-item, clinician-rated, unstructured interview assessing symptom severity over the past week, with each item rated on a 7-point severity scale (1–7) [5]. Higher scores indicate greater severity of schizophrenia symptoms, and the scale comprises five symptom clusters: positive, negative, disorganized, anxious-depressive, and excitement/hostility [6]. | The PANSS has reported good inter-rater reliability (ICC=0.83-0.87). appropriate test-retest reliability (ICC=0.60-0.80), and good internal reliability (α =0.73-83) [5, 7]. |
| Self-esteem | Rosenberg Self-Esteem Scale (RSES) | The RSES is a 10-item self-report questionnaire rated on a Likert scale to measure self-esteem. Each item is scored from 1 to 4, yielding a total score ranging from 10 to 40, with higher scores indicating higher self-esteem and lower scores indicating lower self-esteem [8]. | The RSES has shown an excellent test-retest reliability (ICC=0.93) and a good internal consistency (α=0.75) [8]. |
| Regulation of emotions | Difficulties in Emotion Regulation Scale (DERS-18) | This self-report measure assesses the ability to understand, accept, and manage emotions effectively. It consists of 18 items rated on a Likert scale from 1 to 5, yielding total scores ranging from 18 to 90, with higher scores indicating better emotion regulation and lower scores indicating poorer emotion regulation [9]. | The DERS-18 has an excellent internal reliability in individuals with severe mental disorders, including psychosis (α =0.90) [9]. |
| Quality of life enjoyment and satisfaction | Quality of Life Enjoyment and Satisfaction Questionnaire Short Form (QLES-Q-SF) | The QLES-Q-SF assesses the degree of enjoyment and satisfaction experienced in various areas of daily functioning over the past seven days. It is a self-report measure consisting of 16 items rated on a Likert scale from 1 to 5, yielding total scores ranging from 16 to 80 [10]. | The QLES-Q-SF has demonstrated high internal consistency (α=0.90) and test-retest reliability (ICC=0.93) in a psychiatric population. The questionnaire demonstrated a sensitivity of 80% and a specificity of 100% [10]. |
| Feeling of presence | Igroup Presence Questionnaire (IPQ) | The IPQ assesses the sense of presence experienced in a virtual environment. This self-report scale consists of 14 Likert-type items ranging from 0 to 6, yielding a total score ranging from 0 to 84 [11]. It consists of three subscales: special presence, involvement, and realness [12]. | The IPQ subscales yielded good internal consistency (α=0.63-0.78) in a general population sample [12]. A recent study in a schizophrenia sample found lower internal consistency for the total score (α=0.58), which improved after removing three items (α=0.72) [13]. |
|  | Self-reported sense of presence | Sense of presence was also assessed by asking participants to rate, on a scale from 0 to 10, how much they felt like they were “with the voice” and “discussing with the voice”. The total calculated sense of presence averaged these two items, ranging from 0 to 10. | N/A |
| Adverse and unwanted effects of psychological treatment | Negative Effects Questionnaire (NEQ) | The self-report NEQ assesses adverse and unwanted effects of psychological treatment. It consists of 32 items covering six factors: symptoms (10 items). quality of the treatment (11 items), dependency on the treatment or therapist (2 items), being perceived negatively by others because of undergoing treatment (2 items), hopelessness (4 items), and feelings of incompetence and lowered self-esteem (3 items) [14, 15]. | N/A |

**Table S2**. **Between-group comparison of overall retention, dropouts, exclusions and missing data in participants randomized to complete either virtual reality-assisted therapy (VRT; n=67) or a short course of cognitive behavioral therapy (CBT; n=69).** Fisher tests were preferred over Chi-2 when the expected frequency in any cells of the contingency table was less than 5.

|  | Post-therapy assessment (T2) | | | | 3-month follow-up (T3) | | | | |  |
| --- | --- | --- | --- | --- | --- | --- | --- | --- | --- | --- |
|  | n VRT | n CBT | Test | p-value | n VRT | n CBT | Test | p-value | |  |
| Randomized but did not start therapy | 3 | 1 | Fisher | 0.362 | *N/A* | | | | |  |
| Dropouts | 11 | 9 | Chi-2 | 0.527 | *N/A (no dropouts between T2 and T3)* | | | | |  |
| Exclusions | 3 | 7 | Fisher | 0.327 | *N/A (no exclusions between T2 and T3)* | | | | |  |
| Missing data | 3 | 0 | Fisher | 0.111 | 10 | 4 | Fisher | 0.091 | |  |
| Overall retention | 48 | 53 | Chi-2 | 0.491 | 41 | 49 | Chi-2 | | 0.226 | |

n: number of participants, VRT: virtual reality-assisted therapy, CBT: cognitive behavioral therapy, N/A: non-applicable.

**Table S3. Within-group longitudinal changes following Virtual Reality-assisted Therapy and Cognitive Behavioral Therapy on auditory hallucination severity and related clinical outcomes using linear mixed models (n=136).**

| **Treatment** | | Timepoint values | | | | | | Timepoint comparisons | | | | | | | | | |
| --- | --- | --- | --- | --- | --- | --- | --- | --- | --- | --- | --- | --- | --- | --- | --- | --- | --- |
|  |  | Baseline | | Post-therapy | | 3-month follow-up | | T2-T1 | | | | T3-T1 | | | | | |
|  |  | mean | SD | mean | SD | mean | SD | Cohen's d | 95% CI | p-value | Adjusted p-value | Cohen's d | 95% CI | p-value | Adjusted p-value |  |  |
| **Primary Outcome** | | | | | | | | | | | | | | | | | |
| *Auditory hallucinations (voices)* | | | | | | | | | | | | | | | | | |
| **PSYRATS-AH Total (0-44)** | | | | | | | | | | | | | | | | | |
|  | VRT | 28.8 | 5.0 | 24.1 | 9.3 | 21.5 | 10.3 | **0.796** | **-0.424 - 1.168** | **<0.001** | *N/A* | **1.160** | **-0.762 - 1.558** | **<0.001** | *N/A* |  |  |
|  | CBT | 27.8 | 6.6 | 24.4 | 7.8 | 25.5 | 6.1 | **0.572** | **-0.222 - 0.921** | **0.004** | *N/A* | 0.383 | -0.031 - 0.735 | 0.080 | *N/A* |  |  |
| **Secondary Outcomes** | | | | | | | | | | | | | | | | | |
| *Auditory hallucinations (voices) – Subscales* | | | | | | | | | | | | | | | | | |
| **PSYRATS-AH Distress (0-20)** | | | | | | | | | | | | | | | | | |
|  | VRT | 14.6 | 3.4 | 12.0 | 5.1 | 10.8 | 5.5 | **0.652** | **-0.294 - 1.009** | **0.001** | **0.002** | **0.908** | **-0.540 - 1.275** | **<0.001** | **<0.001** |  |  |
|  | CBT | 14.0 | 4.3 | 12.0 | 4.8 | 12.5 | 4.5 | **0.489** | **-0.151 - 0.827** | **0.012** | 0.047 | 0.359 | -0.029 - 0.690 | 0.080 | 0.160 |  |  |
| **PSYRATS-AH Frequency (0-12)** | | | | | | | | | | | | | | | | | |
|  | VRT | 7.0 | 2.1 | 5.7 | 2.6 | 5.0 | 2.8 | **0.565** | **-0.279 - 0.850** | **<0.001** | **0.001** | **0.802** | **-0.485 - 1.119** | **<0.001** | **<0.001** |  |  |
|  | CBT | 7.1 | 2.1 | 6.4 | 2.4 | 6.3 | 2.1 | **0.323** | **-0.055 - 0.590** | **0.045** | 0.090 | **0.345** | **-0.060 - 0.631** | **0.044** | 0.160 |  |  |
| **PSYRATS-AH Attribution (0-8)** | | | | | | | | | | | | | | | | | |
|  | VRT | 5.3 | 1.8 | 4.4 | 2.2 | 4.1 | 2.5 | **0.453** | **-0.156 - 0.749** | **0.007** | **0.010** | **0.588** | **-0.242 - 0.934** | **0.002** | **0.003** |  |  |
|  | CBT | 4.8 | 2.1 | 4.3 | 2.1 | 4.9 | 2.1 | 0.281 | 0.000 - 0.561 | 0.118 | 0.157 | 0.034 | -0.281 - 0.350 | 0.975 | 0.975 |  |  |
| **PSYRATS-AH Loudness (0-4)** | | | | | | | | | | | | | | | | | |
|  | VRT | 2.0 | 0.9 | 1.9 | 1.0 | 1.6 | 1.0 | 0.083 | -0.271 - 0.436 | 0.889 | 0.886 | **0.484** | **-0.140 - 0.828** | **0.015** | **0.015** |  |  |
|  | CBT | 2.0 | 0.9 | 1.7 | 0.9 | 1.8 | 0.7 | 0.305 | -0.034 - 0.644 | 0.177 | 0.177 | 0.235 | -0.083 - 0.553 | 0.311 | 0.414 |  |  |
| *Delusions* | | | | | | | | | | | | | | | | | |
| **PSYRATS-D Total (0-24)** | | | | | | | | | | | | | | | | | |
|  | VRT | 13.9 | 4.6 | 11.1 | 4.9 | 11.8 | 4.8 | **0.644** | **-0.338 - 0.950** | **<0.001** | **<0.001** | **0.499** | **-0.196 - 0.803** | **0.003** | **0.010** |  |  |
|  | CBT | 12.4 | 4.5 | 11.5 | 4.8 | 11.2 | 3.5 | 0.180 | -0.107 - 0.468 | 0.428 | 0.642 | 0.231 | -0.051 - 0.513 | 0.238 | 0.442 |  |  |
| **PSYRATS-D Distress (0-8)** | | | | | | | | | | | | | | | | | |
|  | VRT | 5.0 | 2.3 | 3.6 | 2.6 | 4.1 | 2.6 | **0.574** | **-0.266 - 0.883** | **0.001** | **0.001** | 0.376 | -0.039 - 0.714 | 0.070 | 0.070 |  |  |
|  | CBT | 4.0 | 2.7 | 4.0 | 2.5 | 3.2 | 2.6 | 0.054 | -0.237 - 0.345 | 0.928 | 0.928 | 0.231 | -0.085 - 0.547 | 0.317 | 0.442 |  |  |
| **PSYRATS-D Frequency (0-16)** | | | | | | | | | | | | | | | | | |
|  | VRT | 8.8 | 2.9 | 7.5 | 3.0 | 7.7 | 3.0 | **0.523** | **-0.213 - 0.833** | **0.003** | **0.003** | **0.462** | **-0.159 - 0.764** | **0.007** | **0.011** |  |  |
|  | CBT | 8.5 | 2.7 | 7.5 | 2.8 | 8.0 | 1.8 | **0.350** | **-0.055 - 0.645** | **0.050** | 0.150 | 0.173 | -0.108 - 0.455 | 0.442 | 0.442 |  |  |
| *Psychotic symptomatology* | | | | | | | | | | | | | | | | |  |
| **PANSS Total (30-120)** | | | | | | | | | | | | | | | | |  |
|  | VRT | 83.0 | 9.5 | 77.6 | 9.7 | 77.0 | 10.7 | **0.626** | **-0.370 - 0.881** | **<0.001** | **<0.001** | **0.624** | **-0.317 - 0.931** | **<0.001** | **0.001** |  |  |
|  | CBT | 81.0 | 7.4 | 75.7 | 9.0 | 75.2 | 9.6 | **0.529** | **-0.286 - 0.772** | **<0.001** | **<0.001** | **0.587** | **-0.302 - 0.871** | **<0.001** | **0.001** |  |  |
| **PANSS Positive (4-28)** | | | | | | | | | | | | | | | | |  |
|  | VRT | 14.3 | 2.4 | 13.2 | 2.3 | 13.0 | 2.3 | **0.497** | **-0.239 - 0.755** | **<0.001** | **<0.001** | **0.542** | **-0.278 - 0.806** | **<0.001** | **0.001** |  |  |
|  | CBT | 14.4 | 1.9 | 13.5 | 2.1 | 13.5 | 1.9 | **0.403** | **-0.157 - 0.650** | **0.003** | **0.007** | **0.424** | **-0.178 - 0.669** | **0.002** | **0.005** |  |  |
| **PANSS Negative (6-42)** | | | | | | | | | | | | | | | | |  |
|  | VRT | 17.4 | 3.6 | 16.6 | 3.4 | 16.0 | 3.7 | 0.296 | -0.046 - 0.546 | 0.051 | 0.076 | **0.385** | **-0.116 - 0.653** | **0.013** | **0.026** |  |  |
|  | CBT | 16.2 | 3.1 | 15.2 | 3.1 | 14.7 | 3.1 | 0.250 | -0.011 - 0.488 | 0.097 | 0.146 | **0.414** | **-0.164 - 0.664** | **0.003** | **0.005** |  |  |
| **PANSS Disorganised (3-21)** | | | | | | | | | | | | | | | | |  |
|  | VRT | 9.6 | 2.0 | 8.8 | 1.8 | 8.9 | 1.7 | **0.328** | **-0.078 - 0.579** | **0.026** | 0.052 | 0.320 | -0.019 - 0.620 | 0.089 | 0.134 |  |  |
|  | CBT | 9.6 | 2.4 | 8.6 | 2.2 | 8.5 | 2.3 | **0.414** | **-0.172 - 0.656** | **0.002** | **0.006** | **0.475** | **-0.193 - 0.757** | **0.003** | **0.005** |  |  |
| **PANSS Excited/Hostility (4-28)** | | | | | | | | | | | | | | | | |  |
|  | VRT | 6.6 | 1.7 | 6.2 | 1.8 | 6.2 | 1.5 | 0.185 | -0.115 - 0.485 | 0.442 | 0.442 | 0.204 | -0.130 - 0.537 | 0.447 | 0.447 |  |  |
|  | CBT | 6.7 | 2.2 | 6.2 | 2.1 | 6.7 | 2.3 | 0.207 | -0.082 - 0.495 | 0.331 | 0.398 | 0.026 | 0.284 - 0.336 | 0.985 | 0.985 |  |  |
| **PANSS Anxio-depressive (3-21)** | | | | | | | | | | | | | | | | |  |
|  | VRT | 7.8 | 2.3 | 7.0 | 2.4 | 7.2 | 2.6 | 0.283 | -0.011 - 0.554 | 0.098 | 0.118 | 0.187 | -0.093 - 0.468 | 0.384 | 0.447 |  |  |
|  | CBT | 7.6 | 2.6 | 7.2 | 2.6 | 7.0 | 2.5 | 0.152 | -0.107 - 0.411 | 0.476 | 0.476 | 0.235 | -0.027 - 0.497 | 0.180 | 0.215 |  |  |
| *Beliefs About Voices* | | | | | | | | | | | | | | | | |  |
| **BAVQR Total (0-105)** | | | | | | | | | | | | | | | | |  |
|  | VRT | 46.5 | 12.9 | 41.0 | 14.7 | 42.3 | 12.4 | **0.381** | **-0.133 - 0.629** | **0.007** | **0.020** | **0.347** | **-0.126 - 0.568** | **0.006** | **0.017** |  |  |
|  | CBT | 47.9 | 15.4 | 45.9 | 14.6 | 45.5 | 11.5 | 0.080 | -0.155 - 0.314 | 0.780 | 0.915 | 0.104 | -0.099 - 0.306 | 0.568 | 0.776 |  |  |
| **BAVQR Malevolence (0-18)** | | | | | | | | | | | | | | | | |  |
|  | VRT | 10.0 | 4.2 | 8.9 | 4.8 | 8.9 | 4.4 | 0.250 | -0.001 - 0.499 | 0.116 | 0.232 | **0.312** | **-0.085 - 0.539** | **0.018** | **0.036** |  |  |
|  | CBT | 10.1 | 4.5 | 8.6 | 5.1 | 8.1 | 4.4 | **0.322** | **-0.083 - 0.561** | **0.021** | 0.128 | **0.448** | **-0.237 - 0.659** | **<0.001** | **0.001** |  |  |
| **BAVQR Benevolence (0-18)** | | | | | | | | | | | | | | | | |  |
|  | VRT | 4.1 | 4.5 | 3.3 | 4.0 | 4.0 | 4.4 | 0.123 | -0.097 - 0.344 | 0.510 | 0.565 | 0.033 | -0.185 - 0.252 | 0.951 | 0.951 |  |  |
|  | CBT | 5.3 | 5.7 | 5.7 | 5.2 | 6.1 | 5.2 | 0.103 | -0.108 - 0.314 | 0.600 | 0.907 | 0.169 | -0.034 - 0.372 | 0.227 | 0.680 |  |  |
| **BAVQR Omnipotence (0-18)** | | | | | | | | | | | | | | | | |  |
|  | VRT | 9.5 | 3.9 | 7.7 | 4.4 | 8.0 | 3.9 | **0.434** | **-0.173 - 0.695** | **0.003** | **0.017** | **0.405** | **-0.151 - 0.660** | **0.005** | **0.017** |  |  |
|  | CBT | 8.5 | 4.1 | 8.1 | 4.1 | 7.9 | 3.5 | 0.050 | -0.197 - 0.296 | 0.915 | 0.915 | 0.080 | -0.153 - 0.313 | 0.776 | 0.776 |  |  |
| **BAVQR Resistance (0-27)** | | | | | | | | | | | | | | | | | |
|  | VRT | 18.0 | 4.9 | 16.8 | 6.0 | 17.1 | 5.2 | 0.180 | -0.086 - 0.445 | 0.375 | 0.563 | 0.142 | -0.120 - 0.404 | 0.531 | 0.637 |  |  |
|  | CBT | 17.5 | 6.9 | 16.4 | 7.2 | 16.4 | 6.0 | 0.124 | -0.130 - 0.379 | 0.598 | 0.907 | 0.110 | -0.132 - 0.351 | 0.643 | 0.776 |  |  |
| **BAVQR Engagement (0-24)** | | | | | | | | | | | | | | | | | |
|  | VRT | 5.0 | 5.5 | 4.2 | 5.1 | 4.2 | 4.9 | 0.116 | -0.109 - 0.342 | 0.565 | 0.565 | 0.132 | -0.100 - 0.364 | 0.497 | 0.637 |  |  |
|  | CBT | 6.6 | 7.1 | 7.2 | 6.5 | 7.0 | 6.8 | 0.105 | 0.112 - 0.321 | 0.605 | 0.907 | 0.096 | 0.120 - 0.311 | 0.656 | 0.776 |  |  |
| *Voices Acceptance and Action* | | | | | | | | | | | | | | | | | |
| **VAAS Total (31-124)** | | | | | | | | | | | | | | | | | |
|  | VRT | 84.9 | 10.0 | 86.9 | 10.8 | 89.8 | 10.9 | 0.130 | -0.099 - 0.359 | 0.499 | *N/A* | **0.389** | **0.121 - 0.657** | **0.012** | *N/A* |  |  |
|  | CBT | 84.4 | 10.9 | 88.0 | 9.4 | 89.0 | 9.1 | **0.350** | **0.128 - 0.572** | **0.005** | *N/A* | **0.442** | **0.191 - 0.692** | **0.002** | *N/A* |  |  |
| *Emotional Regulation* | | | | | | | | | | | | | | | | | |
| **DERS Total (18-90)** | | | | | | | | | | | | | | | | | |
|  | VRT | 48.5 | 12.0 | 44.7 | 11.7 | 42.3 | 11.2 | **0.344** | **-0.105 - 0.583** | **0.012** | *N/A* | **0.498** | **-0.251 - 0.745** | **<0.001** | *N/A* |  |  |
|  | CBT | 45.7 | 10.9 | 43.3 | 12.4 | 42.1 | 11.9 | 0.200 | -0.028 - 0.427 | 0.191 | *N/A* | **0.292** | **-0.067 - 0.517** | **0.028** | *N/A* |  |  |
| *Self-Esteem* | | | | | | | | | | | | | | | | | |
| **RSES Total (0-40)** | | | | | | | | | | | | | | | | | |
|  | VRT | 27.6 | 5.5 | 29.3 | 5.1 | 29.4 | 5.6 | **0.347** | **0.125 - 0.568** | **0.006** | *N/A* | **0.337** | **0.093 - 0.580** | **0.017** | *N/A* |  |  |
|  | CBT | 28.5 | 5.8 | 30.1 | 5.2 | 29.3 | 5.1 | **0.294** | **0.083 - 0.506** | **0.016** | *N/A* | 0.190 | -0.034 - 0.413 | 0.213 | *N/A* |  |  |
| *Quality of Life Enjoyment and Satisfaction* | | | | | | | | | | | | | | | | | |
| **QLESQ-SF Total (14-70)** | | | | | | | | | | | | | | | | | |
|  | VRT | 49.1 | 8.6 | 51.1 | 8.5 | 51.4 | 7.3 | 0.222 | -0.050 - 0.494 | 0.239 | *N/A* | 0.211 | -0.014 - 0.435 | 0.151 | *N/A* |  |  |
|  | CBT | 49.7 | 7.6 | 49.7 | 8.3 | 49.7 | 7.2 | 0.001 | -0.258 - 0.261 | 0.100 | *N/A* | 0.018 | -0.190 - 0.227 | 0.984 | *N/A* |  |  |

**VRT:** Virtual Reality-assisted Therapy**, CBT**: Cognitive Behavioral Therapy, **PSYRATS**: Psychotic Symptoms Rating Scale, **PANSS**: Positive And Negative Syndrome Scale, **BAVQ-R**: Beliefs About Voices Questionnaire-Revised, **VAAS**: Voices Acceptance and Action Scale, **DERS-18**: Difficulties in Emotion Regulation Scale, **RSES:** Rosenberg Self-Esteem Scale, **QLES-Q-SF**: Quality of Life Enjoyment and Satisfaction Questionnaire Short Form. Data are raw mean scores with standard deviation (SD). Linear mixed models with maximum-likelihood estimation were used. Cohen’s d are presented in absolute values. 95% CI: 95% confidence interval for Cohen’s d (min-max values). Significant differences (p-value <0.05) are found in bold. Adjusted *p*-values were calculated using the false discovery rate (FDR) procedure.

**Table S4.** **Protocol deviations related to therapy duration and assessment intervals.** n=136

| Delays | **VRT** | | | | **CBT** | | | | **Between-group comparison** |
| --- | --- | --- | --- | --- | --- | --- | --- | --- | --- |
|  | n | Mean (SD), in days | Min-Max (days) | Protocol deviations (%) | n | Mean (SD), in days | Min-Max (days) | Protocol deviations (%) | p-value |
| Baseline assessment to first therapy session (expected: 7 days) | 65 | 15.2 (23.0) | 4-123 | 9.0 | 65 | 15.4 (22.9) | 4-170 | 1.5 | 0.002 |
| Duration of therapy sessions (expected: 63 days) | 48 | 79.8 (24.8) | 56-172 | 18.9 | 49 | 79.2 (24.2) | 53-232 | 15.7 | 0.931 |
| Last therapy session to post-treatment assessment (expected: 7 days) | 49 | 12.0 (8.0) | 3-50 | 0.0 | 46 | 11.7 (7.1) | 4–34 | 4.2 | 0.387 |
| Last therapy session to 3-month follow-up assessment (expected: 90 days) | 42 | 95.5 (15.5) | 77–156 | 2.0 | 37 | 95.45 (15.5) | 62–148 | 12.2 | 0.723 |

Protocol deviations (%): Percentages are calculated based on the number of participants whose delays exceeded the predefined ±30-day window relative to the scheduled dates. VRT: Virtual Reality-assisted Therapy, CBT: Cognitive Behavioral Therapy. p-values were calculated using Mann-Whitney U tests for independent samples.

**Table S5.** **Protocol deviations related to changes in antipsychotic treatment during the study.** n=135

|  | **VRT** | | | **CBT** | | | **Between-group differences** |
| --- | --- | --- | --- | --- | --- | --- | --- |
|  | n change | n total (available data) | Protocol deviations (%) | n change | n total  (available data) | Protocol deviations (%) | p-value |
| Baseline to post-treatment assessment | 11 | 48 | 22.9% | 14 | 53 | 26.4% | 0.650 |
| Baseline to 3-month follow-up treatment assessment | 21 | 41 | 51.2% | 24 | 49 | 49.0% | 0.842 |

VRT: Virtual Reality-assisted Therapy, CBT: short-course Cognitive Behavioral Therapy. p-values were calculated using Chi-square tests. Any change in the total dose of antipsychotic, measured in olanzapine equivalents, was characterized as a protocol deviation.

**Table S6.** **Equivalent olanzapine dosage at baseline, post-therapy and 3-month follow-up.** n=135

|  |  | Baseline | Post-therapy | 3-month follow-up | Timepoint comparisons | |
| --- | --- | --- | --- | --- | --- | --- |
|  |  |  |  |  | Post-therapy – baseline difference | 3-month follow-up – baseline difference |
| **VRT** | n | 66 | 48 | 41 | 48 | 41 |
|  | Mean (SD) | 22.9 (17.0) | 21.5 (15.5) | 23.1 (16.8) | 0.1 (3.2) | 1.3 (6.4) |
|  | Min-Max | 0.0-75.7 | 0.0- 76.3 | 0.0-76.3 | -14.3-15.5 | -15.0-28.6 |
| **CBT** | n | 69 | 53 | 49 | 53 | 49 |
|  | Mean (SD) | 23.1 (19.9) | 23.2 (21.4) | 24.0 (22.0) | 0.6 (4.7) | 1.0 (5.2) |
|  | Min-Max | 0.0-106.7 | 0.0-106.7 | 0.0-106.7 | -14.6-23.1 | -7.0-19.8 |
| **Between-group**  **differences** | p-value | 0.972 | 0.981 | 0.824 | 0.822 | 0.406 |

VRT: Virtual Reality-assisted Therapy, CBT: Cognitive Behavioral Therapy, N: number of participants. SD: standard deviation. P-values were calculated using Mann-Whitney U tests for independent samples.

**Table S7. Evolution of feeling of presence over the course of Virtual Reality-assisted Therapy as measured using a graphic rating scale and the Igroup Presence Questionnaire.** n=60

| Therapy session | Graphic rating scale (0-10) | | | Igroup Presence Questionnaire (0-84) | | |
| --- | --- | --- | --- | --- | --- | --- |
|  | n | Mean | SD | n | Mean | SD |
| 2 / first | 58 | 7.2 | 2.01 | 60 | 49.1 | 10.58 |
| 3 | 57 | 7.9 | 1.74 |  | | |
| 4 | 56 | 7.7 | 1.92 |  | | |
| 5 | 54 | 7.9 | 1.97 |  | | |
| 6 | 53 | 8.0 | 1.74 |  | | |
| 7 | 51 | 7.5 | 2.22 |  | | |
| 8 | 49 | 8.1 | 2.15 |  | | |
| 9 / last | 46 | 8.0 | 2.15 | 48 | 48.4 | 8.87 |

n: number of participants, SD: standard deviation.

**References**

1.	Shabani A, Masoumian S, Zamirinejad S, Hejri M, Pirmorad T, Yaghmaeezadeh H. Psychometric properties of Structured Clinical Interview for DSM-5 Disorders-Clinician Version (SCID-5-CV). Brain Behav. 2021 May;11(5):e01894. PMID: 33729681. doi: 10.1002/brb3.1894.

2.	Haddock G, McCarron J, Tarrier N, Faragher E. Scales to measure dimensions of hallucinations and delusions: the psychotic symptom rating scales (PSYRATS). Psychological medicine. 1999;29(4):879-89.

3.	Chadwick P, Lees S, Birchwood M. The revised beliefs about voices questionnaire (BAVQ–R). The British Journal of Psychiatry. 2000;177(3):229-32.

4.	Shawyer F, Ratcliff K, Mackinnon A, Farhall J, Hayes SC, Copolov D. The voices acceptance and action scale (VAAS): Pilot data. Journal of clinical psychology. 2007;63(6):593-606.

5.	Kay SR, Fiszbein A, Opler LA. The positive and negative syndrome scale (PANSS) for schizophrenia. Schizophrenia bulletin. 1987;13(2):261-76.

6.	Wallwork R, Fortgang R, Hashimoto R, Weinberger D, Dickinson D. Searching for a consensus five-factor model of the Positive and Negative Syndrome Scale for schizophrenia. Schizophrenia research. 2012;137(1-3):246-50.

7.	Kay SR, Opler LA, Lindenmayer J-P. The positive and negative syndrome scale (PANSS): rationale and standardisation. The British Journal of Psychiatry. 1989;155(S7):59-65.

8.	Muslih M, Chung M-H. Structural validity of the Rosenberg self-esteem scale in patients with schizophrenia in Indonesia. Plos one. 2024;19(5):e0300184.

9.	Charak R, Byllesby BM, Fowler JC, Sharp C, Elhai JD, Frueh BC. Assessment of the revised Difficulties in Emotion Regulation Scales among adolescents and adults with severe mental illness. Psychiatry Res. 2019 Sep;279:278-83. PMID: 30975439. doi: 10.1016/j.psychres.2019.04.010.

10.	Stevanovic D. Quality of Life Enjoyment and Satisfaction Questionnaire-short form for quality of life assessments in clinical practice: a psychometric study. J Psychiatr Ment Health Nurs. 2011 Oct;18(8):744-50. PMID: 21896118. doi: 10.1111/j.1365-2850.2011.01735.x.

11.	Schubert T, Friedmann F, Regenbrecht H. The Experience of Presence: Factor Analytic Insights. Presence: Teleoper Virtual Environ. 2001;10(3):266–81. doi: 10.1162/105474601300343603.

12.	Schubert TW. The sense of presence in virtual environments. Zeitschrift für Medienpsychologie. 2003;15(2):69-71. doi: 10.1026//1617-6383.15.2.69.

13.	Augustin E, Beaudoin M, Giguère S, Ziady H, Phraxayavong K, Dumais A. The Relationship between Sense of Presence, Emotional Response, and Clinical Outcomes in Virtual Reality-Based Therapy for Treatment-Resistant Schizophrenia: An Exploratory Correlational Study. J Pers Med. 2024 Jun 8;14(6). PMID: 38929835. doi: 10.3390/jpm14060614.

14.	Rozental A, Kottorp A, Boettcher J, Andersson G, Carlbring P. Negative effects of psychological treatments: an exploratory factor analysis of the negative effects questionnaire for monitoring and reporting adverse and unwanted events. PloS one. 2016;11(6):e0157503.

15.	Rozental A, Kottorp A, Forsström D, Månsson K, Boettcher J, Andersson G, et al. The Negative Effects Questionnaire: psychometric properties of an instrument for assessing negative effects in psychological treatments. Behavioural and cognitive psychotherapy. 2019;47(5):559-72.
